# Multiomics profiling reveals VDR as a central regulator of mesenchymal stem cell senescence with a known association with osteoporosis after high-fat diet exposure

**DOI:** 10.1038/s41368-024-00309-9

**Published:** 2024-05-22

**Authors:** Jiayao Chen, Shuhong Kuang, Jietao Cen, Yong Zhang, Zongshan Shen, Wei Qin, Qiting Huang, Zifeng Wang, Xianling Gao, Fang Huang, Zhengmei Lin

**Affiliations:** 1 Hospital of Stomatology, Sun Yat-sen University, Guangzhou, China; 2 Guangdong Provincial Key Laboratory of Stomatology, Guangzhou, China; 3 Guanghua School of Stomatology, Sun Yat-sen University, Guangzhou, China; 4 Sun Yat-sen University Cancer Center, State Key Laboratory of Oncology in South China, Collaborative Innovation Center for Cancer Medicine, Guangzhou, China

**Keywords:** Dental diseases, Obesity, Osteoporosis, Stem-cell therapies

## Abstract

The consumption of a high-fat diet (HFD) has been linked to osteoporosis and an increased risk of fragility fractures. However, the specific mechanisms of HFD-induced osteoporosis are not fully understood. Our study shows that exposure to an HFD induces premature senescence in bone marrow mesenchymal stem cells (BMSCs), diminishing their proliferation and osteogenic capability, and thereby contributes to osteoporosis. Transcriptomic and chromatin accessibility analyses revealed the decreased chromatin accessibility of vitamin D receptor (VDR)-binding sequences and decreased VDR signaling in BMSCs from HFD-fed mice, suggesting that VDR is a key regulator of BMSC senescence. Notably, the administration of a VDR activator to HFD-fed mice rescued BMSC senescence and significantly improved osteogenesis, bone mass, and other bone parameters. Mechanistically, VDR activation reduced BMSC senescence by decreasing intracellular reactive oxygen species (ROS) levels and preserving mitochondrial function. Our findings not only elucidate the mechanisms by which an HFD induces BMSC senescence and associated osteoporosis but also offer new insights into treating HFD-induced osteoporosis by targeting the VDR-superoxide dismutase 2 (SOD2)-ROS axis.

## Introduction

The increasing prevalence of a high-fat diet (HFD, ≥30% of energy from fat) has raised concerns about its potential adverse impacts on health,^1,2^ including metabolic comorbidities such as obesity, diabetes, and nonalcoholic hepatitis,^3–7^ as well as its relationship with osteoporosis and bone fragility.^8–10^ HFD feeding results in the development of obesity, which is considered to protect against osteoporosis and osteoporotic fractures because of the positive effect of increased mechanical loading on the skeleton.^11,12^ However, accumulating research evidence shows that this effect is followed by decreased bone formation, bone turnover and an increased risk of fracture resulting from the development of metabolic impairment.^8,13–16^ For example, the dysregulation of lipid metabolism in the body attributed to HFD intake over an extended period can impair bone metabolism, resulting in bone loss and impaired fracture healing.^8–10,17,18^ However, how HFD-driven metabolic dysfunction impacts cellular function in bone tissue has not been determined. In general, a balance between osteoclast-dependent osteoclastogenesis and osteogenic cell-dependent osteogenesis is maintained to maintain bone mass and quality at a steady state.^19,20^ Nevertheless, HFD-induced osteoporosis differs from osteoporosis caused by other factors because it is characterized by reduced osteoclastogenic and osteogenic activity, suggesting that osteogenesis is a potential therapeutic target.^18,21,22^ The specific cellular mechanisms underlying these changes are yet to be fully understood.

Studies have demonstrated the substantial role of bone marrow mesenchymal stem cell (BMSC) senescence in inadequate bone formation associated with osteoporosis,^23,24^ and a decrease in osteogenesis can lead to a decrease in coupled osteoclastogenesis.^25^ BMSCs are mesenchymal stem cells (MSCs) derived from bone marrow with the ability to self-renew and differentiate into osteogenic cells.^26^ Additionally, BMSCs are major regulators of the bone microenvironment. These cells release RANKL, OPG, BMP-2, SFRP2, and other factors that can affect the balance between osteogenesis and osteoclastogenesis and thus are essential for the maintenance of bone homeostasis.^26–29^ However, BMSCs may become senescent due to estrogen deficiency, radiation, and other senescence-inducing stimuli,^30–32^ resulting in a decrease in their properties, such as their proliferation, differentiation capacity, secretion capacity, and homing, and this process plays a key role in the progression of osteoporosis.^33,34^ Based on recent studies reporting that an HFD induces senescence and BMSC dysfunction in HFD-fed individuals,^35–38^ we hypothesize that an HFD induces an accelerated senescence phenotype in BMSCs, contributing to the progression of HFD-induced osteoporosis.

The role of epigenetic regulation, particularly chromatin accessibility remodeling, in the cellular dysfunction of individuals fed an HFD has been widely acknowledged.^39,40^ Chromatin accessibility refers to the extent to which chromatin-binding factors, such as transcription factors (TFs) and RNA polymerase II, can physically interact with chromatinized DNA and is mainly determined by the occupancy and architecture of nucleosomes, which impede access to DNA.^41^ As gene expression is regulated by the binding of TFs to specific DNA sequence motifs across the genome, the chromatin accessibility of these sites to TFs is essential for gene expression. Therefore, changes in the chromatin accessibility of key genes can interfere with fundamental cellular processes, such as cellular metabolism, cell growth, differentiation, and secretion, by disrupting gene expression and thus contribute to the progression of pathologies.^42^ However, the involvement of chromatin accessibility remodeling in BMSCs under HFD feeding has yet to be explored.

The vitamin D receptor (VDR) is a member of the nuclear hormone receptor superfamily and acts as a TF after activation by its ligand, 1,25(OH)_2_D.^43^ Zhou et al. reported that VDR is the key to rejuvenating the osteogenic differentiation of aging MSCs from elderly individuals.^44^ Activated VDR signaling in smooth muscle cells can even induce transdifferentiation into bone-forming-like cells, ultimately leading to matrix calcification.^45^ While VDR is widely known for its role in osteogenesis, interest in its anti-senescence potential continues to increase. Mice deficient in the gene encoding VDR exhibit aging phenotypes,^46^ and VDR activation by 1,25(OH)_2_D can increase the level of Klotho, which protects against aging phenotypes such as skin atrophy, osteopenia, and atherosclerosis.^47^ These findings suggest a potential therapeutic role for VDR in cellular senescence. However, whether VDR signaling plays a role in protecting against HFD-induced BMSC senescence is unclear.

Our study investigated the underlying mechanism of HFD-induced osteoporosis by analyzing the senescence phenotype and intrinsic changes in the transcriptomic and chromatin landscapes of BMSCs from HFD-fed subjects. We revealed that HFD feeding increases the expression of senescence markers that are involved in the transcription of VDR in BMSCs via senescence-associated alterations in chromatin accessibility. Our findings demonstrate that the downregulation of VDR expression is a mediator of BMSC senescence caused by an HFD, thus providing a potential therapeutic strategy for HFD-induced osteoporosis.

## Results

### HFD feeding decreases bone mass and impairs osteogenic activity

To explore the effect of an HFD on bone metabolism, male C57BL/6J mice were fed an HFD (60% kcal from fat) or a normal diet (ND) for 30 weeks. Compared with the ND-fed mice, the HFD-fed mice displayed a 64.03% increase in body weight, greater body size, and greater concentrations of serum triglycerides (TGs) (Fig. S1).

**Fig. 1:**
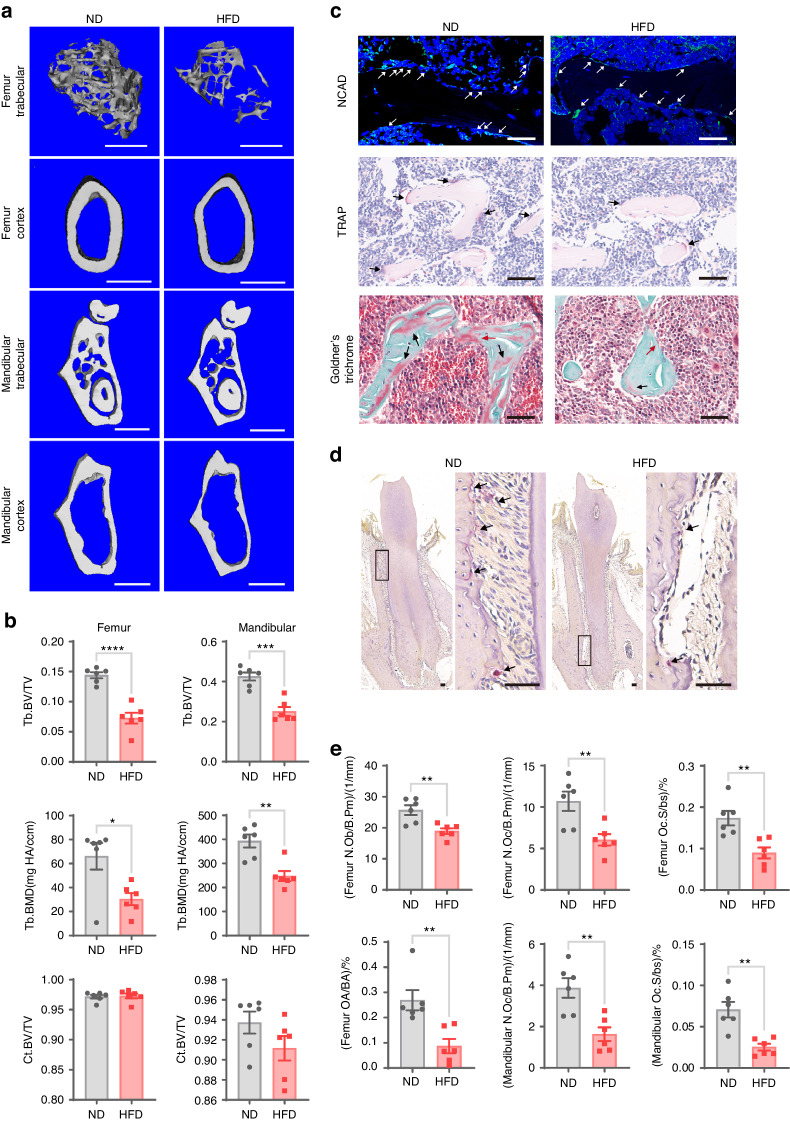
An HFD decreases bone mass and impairs osteoclastogenic and osteogenic activity. **a** Representative 3D-reconstructed μCT images of the trabecular bone in the distal femur metaphysis, the jawbone from the first mandibular molar root furcation region, the cortical bone at the femur mid-diaphysis and the cortical bone from the first mandibular molar root furcation region of ND-fed mice or HFD-fed mice (scale bars = 1 mm). **b** µCT analysis of the trabecular bone volume/total volume (Tb. BV/TV) and BMD in the distal femur trabecular bone or in the first mandibular molar root furcation regions as well as cortical bone volume/total volume (Ct. BV/TV) at the femoral diaphysis or in the first mandibular molar root furcation regions. **c** Representative sections subjected to NCAD immunofluorescence staining, histological TRAP-stained sections, and Goldner’s trichrome-stained sections of the femoral bone marrow cavity from each group (scale bars = 50 μm). NCAD^+^ osteoblasts in the paraffin sections are stained green (white arrows). Osteoclasts in TRAP-stained sections were stained red (black arrow). In Goldner’s trichrome-stained sections, the trabecular surface is covered with intratrabecular (black arrows) and endosteal osteoid layers (red arrows). **d** Representative histological TRAP-stained sections of the alveolar bone of each group (scale bars = 50 μm). **e** Quantification of osteoclastogenic activity and osteogenic activity in the femoral bone marrow cavity or in the alveolar bone of each group (N.Ob/B.Pm osteoblast number per bone perimeter, N.Oc/B.Pm osteoclast number per bone perimeter, Oc.S/bs osteoclast surface per bone surface, OA/BA osteoid area per bone area). The data were shown as the mean ± SEM. *N* = 6, *P < 0.05, **P < 0.01, ***P < 0.001, ****P < 0.000 1

Microcomputed tomography (µCT) analysis revealed a significant reduction in the trabecular bone volume, bone mineral density (BMD), trabecular number, trabecular thickness, and trabecular connectivity density in the distal femur trabecular bone of HFD-fed mice compared to those of ND-fed mice (Fig. 1a, b and Fig. S2A), while there was a significant increase in trabecular separation and the structural model index in the HFD-fed mice (Fig. S2A). Similar decreases were observed in the mandibular trabecular bone parameters of HFD mice (Fig. 1a, b and Fig. S2B). In contrast, cortical bone parameters in neither the mandibular bone nor femur diaphysis showed a significant difference (Fig. 1a, b and Fig. S2C, D). These findings suggest that an HFD acts locally to decrease trabecular bone mass.

Bone mass is the combined result of osteoclastogenic and osteogenic activity. Our study further revealed that expression of the osteoclast marker TRAP in the femur and mandibular bone of HFD-fed mice was repressed (Fig. 1c–e). Additionally, N-cadherin (NCAD) immunofluorescence staining and Goldner’s trichrome staining revealed that both the number of osteoblasts and the amount of new bone formation were decreased in HFD-fed mice (Fig. 1c–e). These findings indicate that a HFD reduces both osteoclastogenic activity and osteogenic activity, leading to repressed bone turnover.

### HFD feeding induces BMSC senescence and suppresses BMSC osteogenesis

**Fig. 2:**
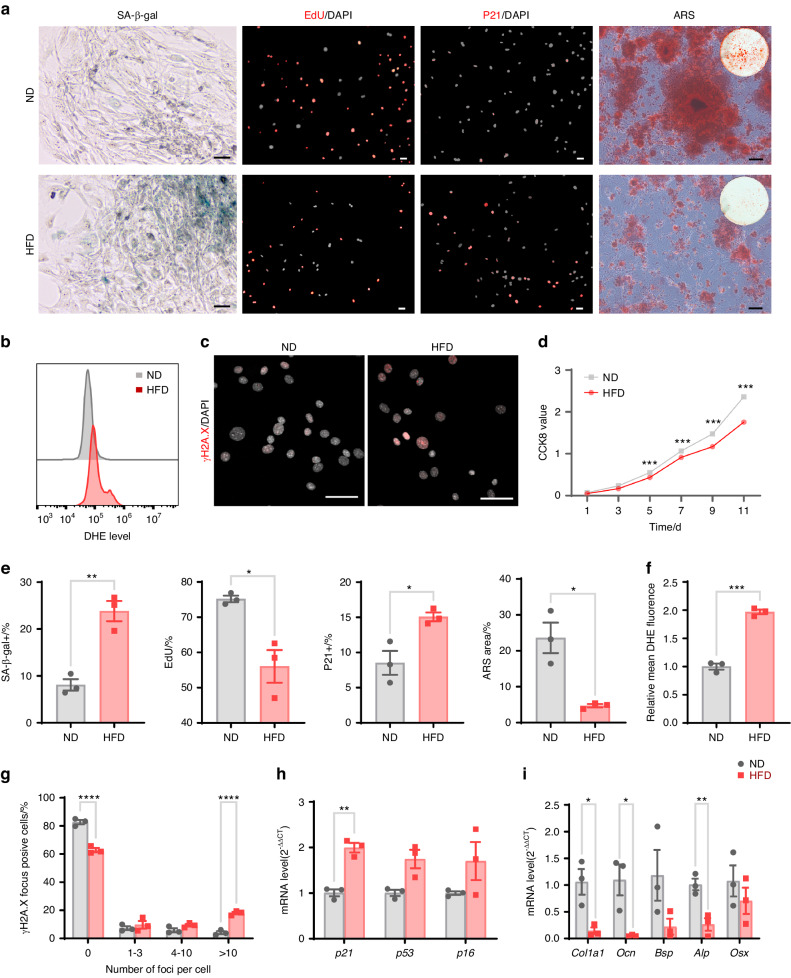
An HFD induces BMSC senescence and suppresses BMSC osteogenesis. **a** Representative images following SA-β-gal, EdU, and p21 immunofluorescence staining and alizarin red staining of BMSCs from HFD-fed mice and ND-fed mice (scale bars = 50 μm). **b** Intracellular ROS levels in BMSCs were detected by DHE staining and flow cytometry. **c** DNA damage markers in BMSCs from HFD-fed mice and ND-fed mice were detected via immunofluorescence staining of γH2A.X (scale bars = 50 μm). **d** CCK8 results showing the proliferative activity of BMSCs from HFD-fed mice and ND-fed mice. **e** Quantification of SA-β-gal, EdU, P21, and ARS staining in the BMSCs from ND- and HFD-fed mice. **f** Statistical analysis of the DHE staining results. **g** Quantification of γH2A.X-positive cell ratios in the BMSCs from ND- and HFD-fed mice. **h** The expression of the cyclin-dependent kinase inhibitors *p21*, *p53*, and *p16* in BMSCs from HFD-fed mice and ND-fed mice was detected by RT‒qPCR. **i** Osteogenesis-related gene expression was quantified by RT‒qPCR after osteogenic induction. The data were shown as the mean ± SEM. *N* = 3, *P < 0.05, **P < 0.01, ***P < 0.001, ****P < 0.000 1

In recent years, accumulating evidence has shown the important role of BMSC senescence in decreasing osteogenic activity.^23,48–50^ Therefore, we sought to investigate whether an HFD could induce senescence in BMSCs. We compared the senescence and proliferation of BMSCs isolated from HFD-fed and ND-fed mice. The results revealed that the activity of senescence-associated markers, including senescence-associated beta-galactosidase (SA-β-gal), mRNA levels of senescence-associated genes (*p21*), percentage of p21^+^ cells, and levels of the DNA damage marker γH2A.X were significantly greater in the BMSCs of HFD-fed mice than in those of ND-fed mice. Additionally, the proliferative capacity of the BMSCs from HFD-fed mice, as measured by the CCK8 assay and EdU staining, was significantly lower than that of the BMSCs from ND-fed mice. Moreover, intracellular reactive oxygen species (ROS) levels, as determined by dihydroethidium (DHE) staining, were significantly greater in BMSCs from HFD-fed mice than in those from ND-fed mice (Fig. 2a–h). Furthermore, BMSCs from HFD-fed mice exhibited impaired osteoblastic differentiation, as evidenced by decreased expression of the osteoblastic genes *Col1a1*, *Ocn*, and *Alp* and decreased numbers of mineralized nodules stained with alizarin red after osteogenic induction (Fig. 2a, h, i).

**Fig. 3:**
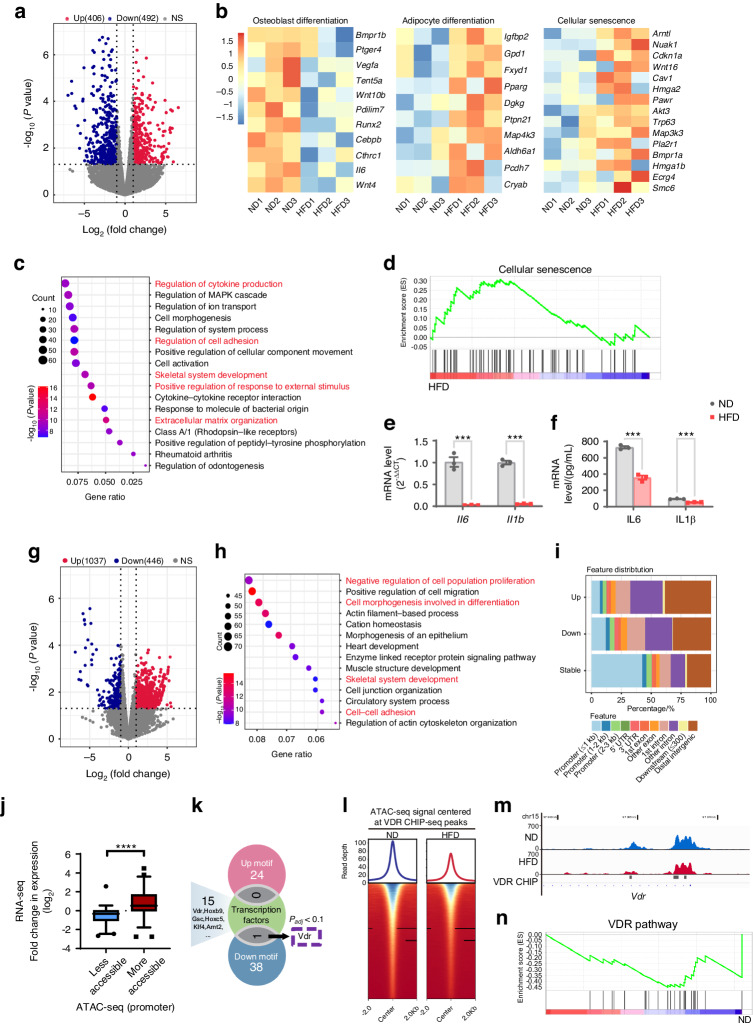
Transcriptome characteristics and chromatin accessibility landscape of BMSCs from HFD-fed mice. **a** Volcano plot showing the expression levels of differentially expressed genes from the BMSCs of HFD-fed mice compared to those from control ND-fed mice. **b** Heatmap showing the downregulated expression of osteogenesis-related genes and upregulated expression of adipogenesis-related genes and senescent genes in BMSCs from HFD-fed mice. **c** Bubble plot of the Gene Ontology (GO) annotation terms enriched in differentially expressed genes in the BMSCs. Terms related to bone development are bolded and highlighted in red. **d** GSEA showing the upregulated expression of senescence-related genes in the BMSCs of HFD-fed mice. **e**, **f** The expression of the proinflammatory genes IL6 and IL1β in BMSCs from HFD-fed mice and ND-fed mice was detected by RT‒qPCR (**e**) and ELISA (**f**), respectively. The data were shown as the mean ± SEM. **g** Volcano plot showing ATAC-seq peaks. Differentially opened/closed peaks are indicated in red/blue for BMSCs from HFD-fed mice compared to those from ND-fed mice as the control. **h** Gene Ontology (GO) analysis of differentially accessible ATAC-seq peaks. Terms related to bone development, cell proliferation, and differentiation are bolded and highlighted in red. **i** Distribution of the genomic features of upregulated, downregulated, and stable ATAC-seq peaks in BMSCs from HFD-fed mice compared to those from ND-fed mice. **j** The differences in chromatin accessibility (ATAC-seq) at the promoter in the HFD group were generally consistent with the differences in expression (RNA-seq). **k** Motif analysis showed that the chromatin accessibility of 24 transcription factor motifs was upregulated, while that of 39 chromatin-binding factor motifs was downregulated in the BMSCs of HFD-fed mice. RNA-seq data revealed 15 transcription factors with differential expression, including *Vdr*, *Hoxb9*, *Gsc*, *Hoxc5*, *Klf4*, and *Arnt2*. After combining the RNA-seq data with the results of motif analysis, we found that there was only one factor, VDR, whose expression and chromatin accessibility to the motif were downregulated (*p*_adj_ < 0.1). **l** Relative chromatin accessibility around VDR ChIP-seq peaks (GSE79813) ± 2 kb. **m** Browser tracks showing downregulated chromatin accessibility at the VDR ChIP-seq peak in BMSCs from HFD-fed mice. **n** GSEA demonstrating the downregulated expression of VDR pathway genes in BMSCs from HFD-fed mice. *N* = 3, *P < 0.05, **P < 0.01, ***P < 0.001

To gain a more comprehensive understanding of the cell-autonomous alterations evoked by an HFD, we conducted RNA sequencing (RNA-seq) of BMSCs isolated from HFD-fed mice and ND-fed mice. Analysis of the RNA-seq data revealed that 898 genes were differentially expressed in BMSCs derived from HFD-fed mice compared to those derived from ND-fed mice, with 406 genes upregulated and 492 genes downregulated (Fig. 3a). RNA-seq analysis of BMSCs revealed the downregulation of key osteogenic genes, including *Wnt4*, *Cthrc1*, and *Runx2*, in the BMSCs of HFD-fed mice (Fig. 3b). Additionally, the expression of key adipocytic genes and ontology gene sets related to cellular senescence was upregulated in the BMSCs from the HFD group compared to those from the ND group (Fig. 3b). Gene Ontology (GO) enrichment analysis indicated that these differentially expressed genes were associated with skeletal system development, cytokine production regulation, cell adhesion regulation, positive regulation of the response to external stimuli, and extracellular matrix organization (Fig. 3c). Gene set enrichment analysis (GSEA) further verified that senescence-related genes were upregulated in the BMSCs from HFD-fed mice (Fig. 3d). These findings suggest that an HFD reduces osteogenesis by inducing senescence, increasing oxidative stress, inhibiting proliferation, and reducing the osteoblastic differentiation capacity of BMSCs.

Unexpectedly, a reduction in the expression of certain classic proinflammatory genes (such as IL6 and IL1β) was observed in BMSCs derived from mice fed an HFD compared to those fed an ND (Fig. S3A). This result was further validated through RT-qPCR and enzyme-linked immunosorbent assay (ELISA) experiments (Fig. 3e, f). Notably, this observation diverges from prior investigations into BMSC senescence induced by alternative factors, such as aging, estrogen deficiency, and radiation, in which the upregulation of these genes has been reported.^31,32,51^ Except for CCR1, no significant alterations were noted in the expression of other chemotaxis receptors (Fig. S3A). These findings imply that the secretory phenotype of senescent BMSCs may exhibit variability under the influence of distinct inducers of cellular senescence, offering a potential explanation for the observed decrease in bone osteoclastogenesis activity in HFD-fed subjects.

### The chromatin accessibility landscape of BMSCs from HFD-fed mice

To gain mechanistic insight into the effects of an HFD on BMSC senescence, we conducted ATAC-seq. This high-throughput method can identify open regions of the genome that are not protected by nucleosomes and are thus accessible to TFs and transcriptionally active. We sequentially extracted RNA and DNA and obtained transcriptome data from RNA-seq as well as open genome regions from ATAC-seq, providing us with an extensive overview of the chromatin landscape responsible for controlling transcription.

Our ATAC-seq data revealed a wide range of changes in chromatin accessibility in BMSCs, suggesting that BMSCs undergo chromatin reprogramming after HFD feeding. We found that 1037 genomic regions were more accessible in BMSCs from HFD-fed mice, while 446 genomic regions became more accessible in BMSCs from ND-fed mice (Fig. 3g). GO enrichment analyses of the genes containing genome regions whose DNA accessibility was altered revealed enrichment in skeletal system development, cell‒cell adhesion, cell proliferation and cell differentiation, etc. (Fig. 3h). The sites that more accessible in BMSCs from mice fed an HFD were observed in distal intergenic regions, while sites less accessible in the BMSCs from mice fed an HFD were enriched in promoters (Fig. 3i). Furthermore, genes with greater accessibility in their promoter regions were found to have significantly greater expression levels (Fig. 3j). Collectively, our findings provide insight into the chromatin accessibility landscape of BMSCs under HFD feeding, which may be a prerequisite for the activation of various genes involved in stemness maintenance and osteogenesis.

ATAC-seq not only allows for the quantification of chromatin region openness but also provides the opportunity to detect potential TF-binding events that may be responsible for chromatin accessibility remodeling and the transcription of nearby genes. To this end, we applied HOMER to identify TF-binding motifs (TFBMs) enriched in differentially accessible genomic regions in BMSCs from HFD-fed or ND-fed mice. We observed 24 TFBMs with increased accessibility and 39 TFBMs with decreased accessibility in BMSCs from HFD-fed mice. RNA-seq data combined with the results of motif analysis revealed VDR as a possible key TF involved in the induction of BMSC senescence, as both its expression and binding motif accessibility were significantly decreased (Fig. 3k). This decrease in chromatin accessibility for VDR-binding sites in BMSCs from HFD-fed mice was further confirmed by ChIP-seq data (GSE79813) (Fig. 3l, m). GSEA of the RNA-seq data revealed the downregulation of VDR target genes in BMSCs from HFD-fed mice (Fig. 3n and Fig. S3B). A decreased VDR expression level was also observed in BMSCs from HFD-fed mice, as evidenced by immunofluorescence staining and RT‒qPCR, further supporting the findings from the RNA-seq data (Fig. S4A‒C). These alterations in gene expression and chromatin accessibility suggest that VDR is a potentially critical regulator of HFD-induced senescence in BMSCs.

### Enhancing VDR signaling can prevent BMSC senescence caused by palmitic acid (PA)

**Fig. 4:**
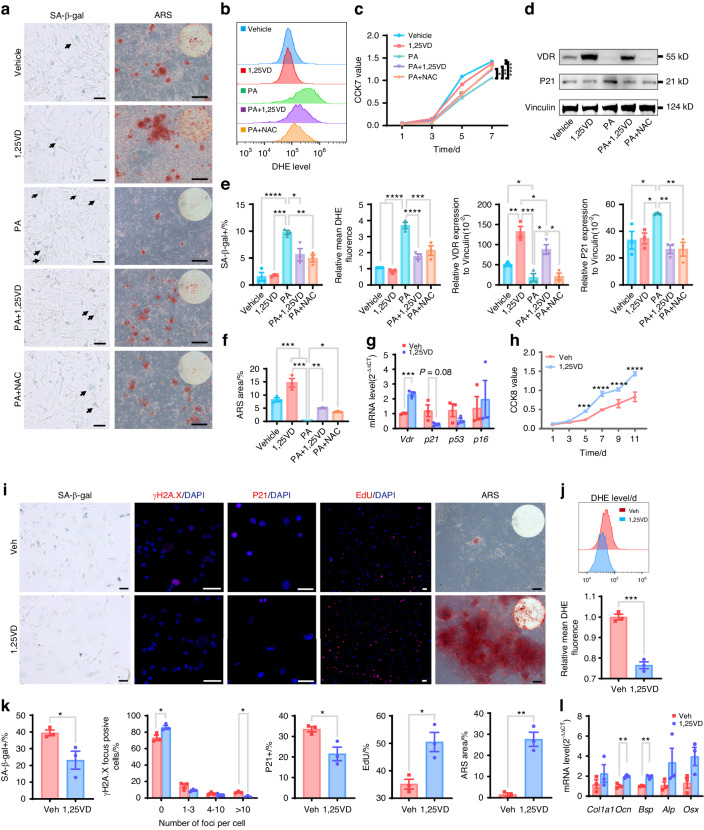
The upregulation of VDR signaling protects BMSCs from senescence caused by palmitic acid or a high-fat diet. **a** Representative images following SA-β-gal staining and alizarin red staining after the osteogenic induction of BMSCs (scale bars = 50 μm). The positive SA-β-gal cells were stained green (black arrow). **b** The addition of 1,25(OH)_2_D or NAC to BMSCs decreased the increase in the intracellular ROS level induced by palmitic acid. ROS levels were analyzed by flow cytometry. **c** The addition of 1,25(OH)_2_D or NAC improved the palmitic acid-induced decrease in the proliferation of BMSCs. Proliferation was analyzed by a CCK8 assay. **d** Western blot results demonstrating the effect of 1,25(OH)_2_D or NAC on VDR and P21 expression in BMSCs. **e** Quantification of the percentage of SA-β-gal-positive cells, ROS levels, and the expression of VDR and P21 in BMSCs from each group. **f** Quantification of ARS staining in BMSCs from each group. **g** The expression of *Vdr*, *p21*, *p53*, and *p16* in BMSCs from mice in the Veh and 1,25VD groups was detected by RT‒qPCR. **h** CCK8 assay demonstrating that the addition of 1,25(OH)_2_D improved the proliferation of BMSCs, which was inhibited by HFD feeding. **i** Representative images of anti-γH2A.X, anti-P21, and EdU immunofluorescence staining as well as SA-β-gal and alizarin red staining in BMSCs from mice in the Veh and 1,25VD groups (scale bars = 50 μm). **j** Intracellular ROS levels, as indicated by DHE staining, in BMSCs that were detected by flow cytometry. **k** Quantification of the SA-β-gal-, γH2A.X-, P21-, EdU-positive cell ratios, and ARS staining in BMSCs from mice in the Veh and 1,25VD groups. **l** Osteogenesis-related gene expression was quantified by RT‒qPCR after osteogenic induction. The data were shown as the mean ± SEM. *N* = 3, *P < 0.05, **P < 0.01, ***P < 0.001, ****P <0.000 1

PA, a major component of an HFD, is often used to recreate the effects of an HFD in in vitro cellular experiments.^52,53^ As expected, compared with BSA-treated BMSCs, PA-treated BMSCs exhibited increased SA-β-gal activity and intracellular ROS levels (Fig. 4a, b, e). Additionally, their proliferative and osteogenic capacities after osteogenic induction were impaired, as shown by the CCK8 assay, measurement of the protein expression of p21, and alizarin red staining (Fig. 4a, c–f). Notably, compared with that in BSA-treated BMSCs, VDR expression in PA-treated BMSCs was lower (Fig. 4d, e). These results suggest that PA is sufficient to simulate HFD-induced senescence in BMSCs in vivo and that VDR may be involved in this process.

To further examine the role of VDR in HFD-induced BMSC senescence, VDR expression was upregulated in cultured BMSCs using the VDR ligand 1,25(OH)_2_D and confirmed by Western blot analysis of VDR protein expression (Fig. 4d, e). Remarkably, senescence hallmarks in BMSCs caused by PA were suppressed after treatment with 1,25(OH)_2_D, as evidenced by the reduced SA-β-gal activity and intracellular ROS levels in the PA + 1,25VD group compared to those in the PA group (Fig. 4a, b, e). Furthermore, compared with BMSCs treated with PA alone, BMSCs treated with PA + 1,25(OH)_2_D also exhibited improved proliferative and osteogenic capacities, as evaluated by a CCK8 assay, measurement of the protein expression of p21 and ARS staining (Fig. 4a, c–f). Therefore, BMSCs with upregulated VDR signaling are capable of resisting PA-induced cellular senescence.

### Activation of VDR signaling can counteract the senescence and bone loss caused by an HFD in BMSCs

Owing to the remarkable resilience of BMSCs with increased VDR signaling to PA-induced senescence in vitro, we proceeded to evaluate whether VDR signaling upregulation could also provide protection in vivo. After HFD-fed mice were administered vehicle or 1,25(OH)_2_D (0.1 µg/kg) intraperitoneally every 3 days for 19 weeks, the phenotypes of the BMSCs, mandible, and femur were analyzed. VDR expression was significantly greater in the BMSCs from 1,25(OH)_2_D-treated mice (1,25VD group) than in those from vehicle-treated mice (Veh group), as demonstrated by immunofluorescence staining and RT‒qPCR (Fig. 4g, Fig. S4D, E). BMSCs obtained from 1,25(OH)_2_D-treated mice exhibited increased proliferative capacity, as measured by a CCK8 assay and EdU staining (Fig. 4h, i, k). Furthermore, senescence hallmarks, such as SA-β-gal activity and levels of the DNA damage marker γH2A.X, were reduced in BMSCs from the 1,25(OH)_2_D-treated mice (Fig. 4i, k). Additionally, the protein level of *p21* was strongly reduced in the BMSCs of the 1,25(OH)_2_D-treated mice, as indicated by the immunofluorescence staining and Western blot results (Fig. 4i, k; Fig. 6h, i). Moreover, supplementation with 1,25(OH)_2_D significantly reduced the intracellular ROS level in BMSCs (Fig. 4j), and improved osteogenic capacity, as evidenced by increased numbers of mineralized nodules stained with ARS and increased mRNA expression of osteoblastic genes (*Ocn*, *Bsp*) after osteogenic induction (Fig. 4i, k, l).

**Fig. 5:**
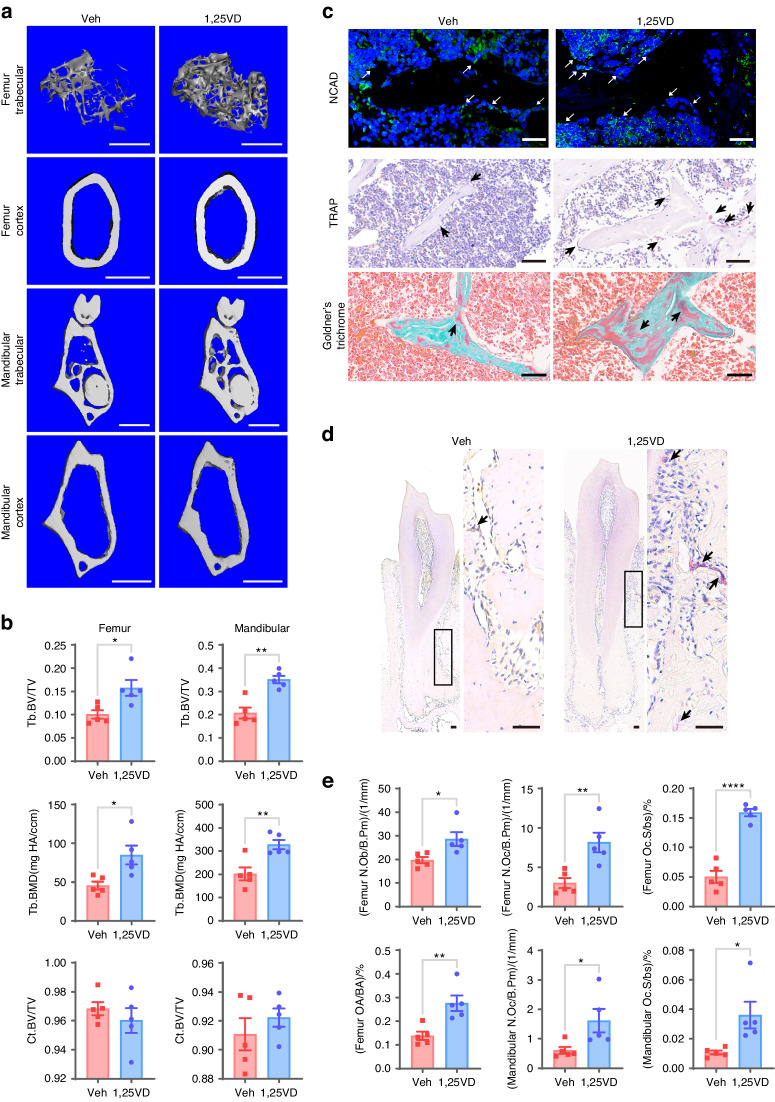
The upregulation of VDR signaling rescues bone loss and decreases osteogenic activity induced by a high-fat diet. **a** Representative 3D-reconstructed μCT images of trabecular bone in the distal femur metaphysis, jaw bone from the first mandibular molar root furcation region, cortical bone at the femur mid-diaphysis and cortical bone from the first mandibular molar root furcation region of the Veh group and 1,25VD group (scale bars = 1 mm). **b** µCT analyses of the trabecular bone volume/total volume (Tb. BV/TV) and BMD in the distal femur trabecular bone or in the first mandibular molar root furcation regions as well as the cortical bone volume/total volume (Ct. BV/TV) at the femoral diaphysis or in the first mandibular molar root furcation regions. **c** Representative sections subjected to NCAD immunofluorescence staining, histological TRAP-stained sections, and Goldner’s trichrome-stained sections of the femoral bone marrow cavity from each group (scale bars = 50 μm). NCAD^+^ osteoblasts in the paraffin sections were stained green (white arrows). Osteoclasts in the TRAP-stained sections were stained red (black arrow). In Goldner’s trichrome-stained sections, the trabecular surface was covered with intratrabecular osteoid (black arrows). **d** Representative histological TRAP-stained sections of the alveolar bone of each group (scale bars = 50 μm). **e** Quantification of osteoclastogenic activity and osteogenic activity in the femoral bone marrow cavity or in the alveolar bone of each group (N.Ob/B.Pm osteoblast number per bone perimeter, N.Oc/B.Pm osteoclast number per bone perimeter, Oc.S/bs osteoclast surface per bone surface, OA/BA osteoid area per bone area). The data were shown as the mean ± SEM. *N* = 5, *P < 0.05, **P < 0.01, ***P < 0.001, ****P < 0.000 1

We further investigated whether supplementation with 1,25(OH)_2_D could reduce the bone loss caused by an HFD. µCT analysis revealed significant increases in the trabecular bone volume, BMD, trabecular number, and trabecular connectivity density, as well as decreases in the trabecular separation within the distal femur metaphysis of mice treated with 1,25(OH)_2_D compared to those of mice treated with the vehicle (Fig. 5a, b and Fig. S5A). Similar trends were evident in the trabecular parameters of the mandibular bone isolated from 1,25(OH)_2_D-treated mice (Fig. 5a, b and S5B). Additionally, a substantial increase in cortical bone thickness in the femur was noted in the 1,25(OH)_2_D-treated mice compared to that in the vehicle group (Fig. S5C). Conversely, other cortical bone parameters in both the mandible and femur diaphysis exhibited no statistically significant difference (Fig. 5a, b and Fig. S5C, D). Histological analyses of the femur and mandibular sections by TRAP staining indicated an increase in osteoclast activity, while NCAD immunofluorescence staining and Goldner’s trichrome staining of proximal femur sections demonstrated an increase in osteogenic activity, as indicated by increases in osteoblasts and new bone formation, respectively (Fig. 5c–e). Taken together, these results suggest that the upregulation of VDR signaling can protect BMSCs from senescence, restore impaired osteogenic capacity and ameliorate bone loss induced by an HFD.

### BMSC senescence caused by PA is mediated by the accumulation of ROS

Previous studies have demonstrated that VDR signaling has antioxidant capacity by upregulating NRF2, FOXO3, and other regulators of antioxidant pathways.^54,55^ Consistently, our results showed enhanced resistance to the upregulation of ROS levels induced by HFD feeding or its components in BMSCs treated with activators of VDR signaling. To determine if HFD-induced BMSC senescence was caused by the accumulation of ROS, we compared the senescence phenotypes of BMSCs treated with PA + NAC to those supplemented with PA or PA + 1,25(OH)_2_D. Compared to PA alone, PA + NAC greatly reduced SA-β-gal activity and p21 protein expression and improved proliferation and osteogenic capacity to a level comparable to that of PA + 1,25(OH)_2_D treatment (Fig. 4a, c–f). Additionally, a previous in vivo study revealed that an HFD impairs the proliferative property of BMSCs by increasing ROS levels, which is in line with our findings.^56^ Moreover, there was no change in VDR protein expression in BMSCs treated with PA + NAC (Fig. 4d, e). Thus, these findings suggest that accumulating ROS play a critical role in PA-induced BMSC senescence, and the anti-senescence effects of NAC supplementation were almost as effective as the effects observed with the upregulation of VDR signaling induced by 1,25(OH)_2_D supplementation.

### Superoxide dismutase 2 (SOD2) is a downstream transcriptional target of VDR that mediates HFD-induced BMSC senescence

**Fig. 6:**
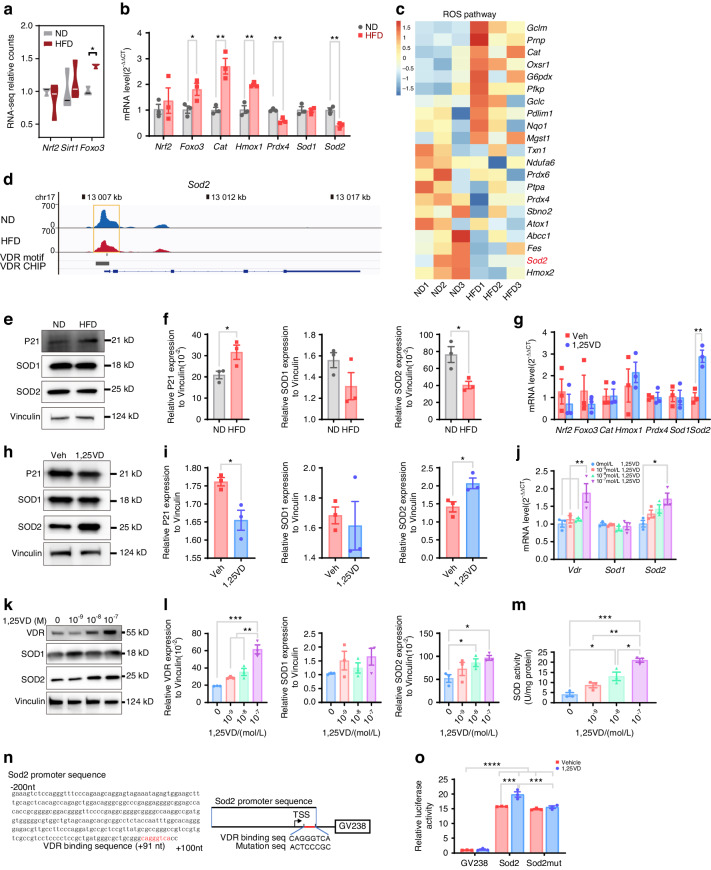
SOD2 is a downstream molecule of VDR signaling in BMSCs. **a** The expression of master regulators of antioxidant pathways, such a*s Nrf2*, *Sirt1*, and *Foxo3*, in BMSCs from ND-fed mice or HFD-fed mice was analyzed via RNA-seq. Violin plots showing the medians, quartiles, and 95% confidence intervals. **b** The expression of *Nrf2*, *Foxo3*, *Cat*, *Hmox1*, *Prdx4*, *Sod1*, and *Sod2* in BMSCs from ND-fed mice or HFD-fed mice was analyzed by RT‒qPCR. **c** Heatmap showing the expression of ROS pathway genes in the BMSCs of ND-fed mice or HFD-fed mice. **d** Browser tracks showing downregulated chromatin accessibility at the VDR ChIP-seq peak and at the VDR motif in the promoter region of *Sod2*. **e** The expression of p21, SOD1, and SOD2 in BMSCs from ND-fed mice or HFD-fed mice was analyzed by Western blot. **f** Statistical analysis of the data in (**e**). **g** The expression of *Nrf2*, *Foxo3*, *Cat*, *Hmox1*, *Prdx4*, *Sod1*, and *Sod2* in BMSCs from mice in the Veh and 1,25VD groups was analyzed by RT‒qPCR. **h** The expression of p21, SOD1, and SOD2 in BMSCs from mice in the Veh and 1,25VD groups was analyzed by Western blot. **i** Statistical analysis of the data in (**h**). **j** The effects of different concentrations of 1,25(OH)_2_D on the mRNA levels of VDR, SOD1, and SOD2 in BMSCs were analyzed via RT‒qPCR. **k** The effects of different concentrations of 1,25(OH)_2_D on the expression of the p21, SOD1, and SOD2 proteins in BMSCs were detected via Western blot. **l** Statistical analysis of the Western blot results. **m** Total SOD enzyme activity data show the effects of different concentrations of 1,25(OH)_2_D on total SOD enzyme activity in BMSCs. **n** The figure shows the VDR-binding sequence (VDR-binding sequence, highlighted in red) in the promoter region of the *Sod2* gene and a simplified diagram of the structure of the Sod2-GV238 plasmid and Sod2mut-GV238 plasmid. **o** Dual-luciferase reporter assay demonstrates that the addition of 1,25(OH)_2_D significantly enhanced luciferase activity driven by the *Sod2* promoter but failed to enhance luciferase activity driven by the *Sod2* promoter with a mutated VDR-binding sequence. *N* = 3, *P < 0.05, **P < 0.01, ***P < 0.001, ****P < 0.000 1

Having established that an HFD reduces VDR signaling, leading to excessive ROS accumulation and BMSC senescence, we next investigated the downstream mechanism through which VDR signaling downregulation mediates increased ROS levels. Our initial hypothesis was that VDR target genes, such as NRF2 and SIRT1/FOXO3,^54,55^ would be downregulated. However, RNA-seq revealed that *Foxo3* was significantly upregulated, while *Nrf2* and *Sirt1* did not change in expression in BMSCs from HFD-fed mice (Fig. 6a). RT‒qPCR also revealed an increase in the mRNA level of *Foxo3* and its downstream antioxidant effectors *Cat* and *Hmox1* (Fig. 6b). We then focused on the expression patterns of genes involved in the ROS pathway, which are oxidative stress response genes. These genes, including the SOD, PRNP, and OXSR1 genes, are essential for ROS metabolism and provide protection against oxidative damage when activated by accumulating ROS.^57–59^ Although numerous antioxidant genes, including *Cat*, *Gclm*, and *G6pdx*, were upregulated in the BMSCs from HFD-fed mice, a decrease in the expression of other antioxidant genes was also noted, with *Sod2* demonstrating the strongest downregulation (Fig. 6c). As a prominent part of the antioxidant system, SOD2 plays a vital role in preventing ROS-induced cellular senescence.^60,61^ RT‒qPCR (Fig. 6b) and Western blot analysis (Fig. 6e, f) indicated a decrease in SOD2 expression upon exposure to an HFD, while SOD1 expression remained unchanged. The upregulation of VDR signaling in HFD-fed mice increased *Sod2* expression (Fig. 6g), and this increase was further confirmed at the protein level (Fig. 6h, i). Due to the pivotal role of CAT in regulating oxidative stress,^62^ we also examined variations in CAT enzymatic activity in BMSCs obtained from mice fed an ND or HFD. However, CAT enzymatic activity exhibited no significant difference between the two groups (Fig. S6A). Our findings suggest that SOD2 may be a key molecular mediator of HFD-induced BMSC senescence through the VDR-ROS axis.

We next sought to determine whether SOD2 is a downstream target of VDR. ATAC-seq revealed a decrease in the enrichment of open chromatin regions near the *Sod2* transcriptional start site, corresponding to the VDR motif and VDR-binding sites (Fig. 6d). Both RT‒qPCR and Western blot analysis demonstrated concurrent increases in the expression levels of SOD2 and VDR corresponding with increasing 1,25(OH)_2_D concentrations (Fig. 6j–l). The addition of 1,25(OH)_2_D resulted in an increase in total SOD activity (Fig. 6m). Furthermore, luciferase activity in cells transfected with the Sod2-GV238 plasmid increased with 1,25(OH)_2_D treatment, while no significant change was observed in cells transfected with the Sod2mut-GV238 plasmid (Fig. 6n, o). To further corroborate these findings, we conducted SOD2-small interfering RNA (si*Sod2*) interference experiments. First, we assessed the efficacy of si*Sod2*. Compared to those of BMSCs transfected with siNC, the SOD2 protein level was lower in BMSCs transfected with si*Sod2* (Fig. S6B, C). Remarkably, when VDR protein levels remained relatively unchanged, the downregulation of SOD2 expression significantly impaired the ability of 1,25(OH)_2_D to decrease intracellular ROS levels, alleviate the senescence phenotype, promote proliferation and enhance osteogenic differentiation capacity (Fig. S6D–H). Moreover, we evaluated the effect of SOD2 loss on the mitochondrial membrane potential using the fluorescent cationic dye JC-1. JC-1 labels mitochondria with high membrane potential red (JC-1 aggregates) and mitochondria with low membrane potential green (JC-1 monomers).^63^ Quantitative analysis through flow cytometry experiments revealed that compared with siNC+PA group, a notable increase in the red fluorescence in siNC+PA + 1,25VD group, which was accompanied by a decrease in the green fluorescence (Fig. S6I, J). Conversely, a contrasting trend emerged upon the downregulation of SOD2 expression (Fig. S6I, J), confirming mitochondrial dysfunction attributable to the loss of SOD2 regulated by VDR signaling. However, the increase in ROS levels induced by PA or SOD2 ablation did not lead to significantly increased BMSC apoptosis, as determined by measuring the levels of the apoptotic markers apoptosis-inducing factor (AIF), cytochrome c and cleaved caspase-3 (Fig. S6K, L). In summary, this collective evidence supports the notion that SOD2 is a crucial downstream target of VDR that is involved in HFD-induced increases in ROS levels and senescence in BMSCs.

## Discussion

The consumption of an HFD has been shown to have a detrimental effect on bone mass and quality, with BMSC dysfunction playing a key role.^37^ Our study has demonstrated that HFD consumption leads to the senescence of BMSCs and impairs their proliferative and osteogenic abilities. By combining ATAC-seq and RNA-seq data, we have provided a comprehensive overview of transcriptomic and chromatin accessibility changes in BMSCs from HFD-fed mice compared to those from ND-fed mice. This has revealed a dynamic transcriptional program and the chromatin landscape of genes related to cellular senescence in BMSCs following HFD feeding. Our data suggest that the downregulation of VDR plays a critical role in the chromatin accessibility alterations associated with BMSC senescence and that VDR is a key regulator of senescence resistance to HFD feeding through its ability to reduce ROS levels by activating SOD2 transcription in BMSCs.

Numerous studies have revealed that an HFD is connected to a host of chronic diseases that originate from cellular senescence.^64,65^ For example, research has revealed that an HFD can cause renal tubular cells to become senescent and secrete proinflammatory cytokines, such as IL1α, TNF-α and MCP-1, resulting in renal fibrosis and impaired renal function.^66^ In addition, Chen et al.^36^ suggested that the senescence of hepatocytes caused by an HFD is a major factor in the development of nonalcoholic fatty liver disease (NAFLD). Our study also revealed that, compared with those from ND-fed mice, BMSCs from HFD-fed mice had increased expression of senescence markers, such as SA-β-gal, γH2A.X, and p21, as well as decreased proliferative capacity, as measured by a CCK8 assay and EdU staining, and osteoblast differentiation potential. BMSCs are essential for bone tissue development and the maintenance of bone strength, as they can self-renew and differentiate into osteoblasts, as well as create an osteogenic microenvironment through the release of pro-osteogenic factors.^26^ However, the senescence of these cells can disrupt bone tissue homeostasis through alterations in cell cycle fractions and the secretion of senescence-associated factors, which negatively impact osteogenesis; thus, senescence is the primary cause of bone loss and the deterioration of bone quality.^67^ Consequently, our findings imply that further investigation into the mechanisms of HFD-induced BMSC senescence may provide useful insights into the treatment of HFD-induced bone deterioration.

Our combined ATAC-seq, RNA-seq, and ChIP-seq analyses revealed that VDR is a key regulator of BMSC senescence under HFD conditions. VDR belongs to the nuclear hormone receptor superfamily and is activated by 1,25(OH)_2_D, the biologically active form of vitamin D, which allows it to translocate to the nucleus and exert its transcriptional activity.^68^ It is primarily known for its role in the regulation of osteogenesis and serves as a mediator of the expression of genes that are vital to osteoblastic identity in early osteoblastic cells as well as in MSCs through coordination with the actions of RUNX2 and C/EBPb.^69^ Our study also revealed that the osteogenic ability of BMSCs was enhanced after activation of the VDR pathway in vitro, confirming the participation of VDR in promoting the osteoblastic differentiation of BMSCs. Notably, studies have shown that VDR is also associated with various aging-related diseases, such as Alzheimer’s disease, sarcopenia, and skin aging.^70–72^ Moreover, Keisala et al.^73^ reported that 6-month-old VDR knockout mice exhibited the features of aging, including a decreased survival rate, early alopecia, epidermal cyst development, and enlarged sebaceous glands. This finding has sparked further research into the anti-senescence effects of VDR, which has been demonstrated to reduce senescence phenotypes in neural stem cells, epithelial cells of the skin, muscle cells, and more.^54,71,74^ Our experiments have confirmed this anti-senescence effect by showing that VDR signaling not only was reduced during BMSC senescence under high-fat conditions but also helped BMSCs resist senescence both in vitro and in vivo. The ability of increased VDR to rescue BMSC senescence in a high-fat microenvironment suggests that the reduced expression of VDR is one of the drivers of BMSC senescence caused by an HFD.

Taken together, our findings suggest that supplementation with 1,25(OH)_2_D, an activator of VDR, not only inhibits BMSC senescence but also reduces the accumulation of ROS levels in BMSCs induced by HFD. Physiologically, ROS plays an important role in regulating stem cell activities by mediating biological signaling through their potent oxidation capacity, maintaining a balance between cell quiescence, proliferation, and differentiation.^75^ However, supraphysiological concentrations of ROS, which are often observed in senescent cells, can lead to the unexpected oxidation of all classes of macromolecules, resulting in mitochondrial dysfunction, genomic instability, lipofuscin accumulation, the denaturation and aggregation of proteins, and the impairment of cell cycle entry.^76^ To address this, we tested the effects of supplementation with the antioxidant NAC on BMSC senescence, and NAC was found to reduce ROS levels and rescue senescence, stimulating cell proliferation and osteoblastic differentiation. Our results imply that the downregulation of VDR expression caused by an HFD mainly induces BMSC senescence by disrupting redox balance.

The intracellular concentration of ROS is maintained under strict control to ensure redox balance.^75^ An HFD can increase ROS production due to excessive food intake, leading to increased formation of the superoxide radical anion (O2·-) from aerobic respiration and electron leakage.^77^ To counter the harmful effects of elevated ROS, a compensatory antioxidant response is crucial to resist stress.^78^ However, a decrease in the expression of SOD2 was observed in the BMSCs of mice subjected to HFD feeding. SOD2 is important for the elimination of excessive mitochondrial O2·-, while SOD1 removes O2·- from the cytoplasm, thereby ensuring the maintenance of redox balance.^79^ Previous studies have established a link between increased ROS levels and cellular senescence due to SOD2 deficiency. For instance, Schoppa et al.^80^ reported that a decrease in osteoblast activity and an increase in osteoblast senescence were caused by a lack of SOD2. This deficit leads to the accumulation of ROS in mitochondria, which can cause damage to macromolecules and induce the release of ROS from the mitochondria into the cytosol, resulting in cellular senescence.^60,81,82^ Consistent with these findings, our study showed that SOD2 expression was reduced and that cellular senescence was increased in BMSCs from mice fed an HFD. Conversely, increased SOD2 levels were observed in BMSCs treated with 1,25(OH)_2_D, accompanied by a reduction in BMSC senescence. This finding suggests that BMSC senescence induced by a HFD is closely related to SOD2 levels. Furthermore, we observed a decrease in the chromatin accessibility of the *Sod2* promoter containing the VDR motif in BMSCs from mice fed an HFD, as well as increases in SOD2 expression and the luciferase activity of the Sod2-GV238 plasmid with the upregulation of VDR signaling in BMSCs. Decreased SOD2 levels significantly delayed the anti-senescence effects of VDR activation in BMSCs. Therefore, we can conclude that redox imbalance in BMSCs induced by the downregulation of VDR signaling is partially due to the reduced activation of SOD2. In addition, the expression of SOD1 and CAT, which are crucial antioxidant molecules, did not correspondingly increase in a high-ROS environment. These findings suggest that an inadequate antioxidant response may also contribute to an HFD-induced increase in ROS, but this hypothesis needs to be clarified in future studies.

An HFD may lead to downregulated VDR signaling in BMSCs through various mechanisms. Han et al.^83^ found that mice fed an HFD exhibited reduced circulating levels of 25-hydroxyvitamin D (25(OH)D), which is a substrate in the conversion to 1,25-dihydroxyvitamin D (1,25(OH)_2_D). This could be attributed to the increased uptake and storage of vitamin D metabolites, including 25(OH)D and 1,25(OH)_2_D, by expanding adipose tissues or to the decreased production of 25(OH)D due to decreased 24-hydroxylase activity.^84,85^ Additionally, VDR expression was significantly downregulated in BMSCs after treatment with PA in vitro, suggesting that reduced 1,25(OH)_2_D levels are not the only possible cause of this difference. Another potential mechanism by which an HFD could reduce VDR signaling is through activation of the RAS/MAPK axis, which is known to downregulate VDR expression.^86^ Our analysis of RNA-seq data revealed regulation of the MAPK cascade in BMSCs from mice fed an HFD. However, further studies are needed to identify the key upstream driver of HFD-induced VDR signaling downregulation.

This work has several limitations that require consideration. According to other studies, HFD consumption for different durations can lead to changes in cell proliferation, differentiation, and secretion.^18,37^ For example, HFD feeding for 8 weeks in mice induces an increase in osteoclast activity, while HFD feeding for 24 weeks induces a significant decrease in osteoclast activity.^18^ Therefore, the ability of an HFD to induce senescence in BMSCs may vary with the feeding duration, and further studies are needed to clarify the temporal relationship between an HFD and BMSC senescence. Another limitation relates to the intervention time and duration of 1,25(OH)_2_D treatment, which may result in different therapeutic effects. Therefore, subsequent studies evaluating the effects of these factors on BMSC senescence and bone metabolism are needed to obtain additional evidence on the role of VDR activation in HFD-induced osteoporosis.

Our analysis of the genome-wide chromatin accessibility landscape of primary BMSCs revealed that reduced VDR signaling plays a pivotal role in HFD-induced BMSC senescence. Furthermore, we found that SOD2 expression is significantly decreased, resulting in increased ROS levels. Consequently, we suggest that targeting VDR signaling may be a potential therapeutic approach for HFD-induced BMSC senescence as well as osteoporosis and other HFD-related diseases. Moreover, our ATAC-seq data provide valuable insight into the binding of TFs and chromatin regulation, which may explain changes in cellular functions and disease progression.

## Materials and methods

### Animals and treatment

The 5-week-old male C57BL/6J mice used in this study were purchased from GemPharmatech. For the HFD mouse model, the mice were randomly divided into an HFD group and an ND group, with 6 mice in each group. Starting from the 5th week of age, the regular feed of mice in the HFD group was replaced with an HFD (D12492; Research Diet), while the ND group mice were consistently fed an ND until they were euthanized at 35 weeks of age. All animals were housed under standard conditions (an indoor temperature of 21 °C, relative humidity of 55%, and a 12-h light–dark cycle). All the experiments were conducted at Sun Yat-sen University with the approval of its Institutional Animal Care and Use Committee (SYSU-IACUC-2018-000269).

For the 1,25(OH)_2_D administration model, the mice were switched from a regular diet to an HFD starting at age 5 weeks. At 16 weeks of age, the mice were randomly divided into two groups: the HFD + 1,25(OH)_2_D administration group (1,25VD) and the HFD + blank administration group (Veh), with five mice in each group. 1,25(OH)_2_D powder (AbMole) was dissolved in corn oil (Aladdin) to a concentration of 0.5 µg/mL. The mice in the 1,25VD group were intraperitoneally injected with the 1,25(OH)_2_D solution every 3 days at a dose of 0.1 µg/kg per injection. The mice in the Veh group received intraperitoneal injections of the same volume of corn oil (vehicle) every 3 days. Tissue samples were collected in the 19th week after administration. All animals were housed under standard conditions (an indoor temperature of 21 °C, a relative humidity of 55%, and a 12-h light–dark cycle). All the experiments were conducted at Sun Yat-sen University with the approval of the Institutional Animal Care and Use Committee (SYSU-IACUC-2021-000637).

### Measurement of serum TGs

After the mice were euthanized, whole-blood samples were collected via the tail vein into regular centrifuge tubes. The tubes were then placed at 4 °C and allowed to clot for 2 h. Afterward, the samples were centrifuged at 2 000 × *g* for 5 min to obtain the serum supernatant. A liquid sample TG assay kit (Applygen Technologies, Inc.) was used according to the manufacturer’s instructions. The samples were incubated at 37 °C for 15 min in a water bath. A microplate reader (BioTek) was used to measure the absorbance at a wavelength of 550 nm. A standard curve was generated based on the OD values obtained from the blank tube and the standard samples, allowing for the calculation of TG levels in the serum samples.

### µCT

The mandible and femur samples were fixed using 4% paraformaldehyde (Wuhan Servicebio Technology) for 24 h. Subsequently, µCT analysis was performed using a µCT scanner (SCANCO Medical). Three-dimensional reconstructions of the mandible and femoral bone were generated using Mimics Research 20.0 software. µCT 50 SCANCO MEDICAL software was used to analyze and measure the bone parameters.

### Histology staining

Mandible and femur samples were fixed in 4% paraformaldehyde for 24 h. After rinsing with PBS 2-3 times, the samples were decalcified with Tri-EDTA decalcification solution (Wuhan Servicebio Technology) for 3 weeks. The samples were embedded in paraffin for the following section. Paraffin sections (4 μm thick) were subjected to TRAP staining (Sigma), immunofluorescence staining, or Goldner’s trichrome staining (Wuhan Servicebio Technology) according to the provided instructions. TRAP-stained sections and Goldner’s trichrome-stained sections were observed under a light microscope (Leica). For the Goldner’s trichrome-stained sections, unmineralized osteoid stained red, and mineralized bone stained green. The osteoid area and mineralized area of the cortical bones in the femurs were quantified using ImageJ software (National Institutes of Health). The ratio of the osteoid area to the total bone area was calculated for three randomly chosen areas throughout each bone section for each sample, as described by Yuan et al.^87^

For immunofluorescence staining, paraffin sections were deparaffinized, antigen repaired with sodium citrate, permeabilized in PBST (1x PBS, 0.2% Triton X-100) for 10 min at room temperature and incubated in sheep serum for blocking. Primary antibodies were incubated with the sections overnight at 4 °C, followed by incubation with secondary antibodies overnight at 4 °C. Nuclei were counterstained with DAPI. Images were acquired using a confocal microscope (Olympus). The primary antibody used in this study was an anti-NCAD antibody (Zen-Bioscience) at a 1:50 dilution. Alexa Fluor-coupled secondary antibodies were used (Cell Signaling Technology) at a dilution of 1:500.

### BMSC isolation and culture

BMSCs were isolated from the HFD, ND, Veh, and 1,25VD groups as described by Tencerova et al.^88^ with slight modifications. After the mice were euthanized, the limbs were dissected, after which the bones were removed and then washed with PBS. Using ophthalmic scissors, the ends of the bone epiphyses were trimmed, and the bone marrow tissue was flushed from the bone marrow cavity. The bones without bone marrow were cut into bone fragments measuring 1–3 mm^2^, which were subsequently digested with a digestion solution containing 1 mg/mL type II collagenase (Gibco) prepared in DF12 medium (Gibco) in a 37 °C shaking incubator at 180 r/min for 5 h. After digestion, the bone pieces were transferred to sterile culture dishes and cultured in a complete medium consisting of DF12 medium and 10% FBS (Gibco). The medium was changed every three days until the cells reached 80% confluence. The adherent cells were dissociated into single cells using TrypLE (Gibco). After centrifugation at 1 000 r/min for 5 min, the supernatant was removed, and 100 μL of PBS was added to gently resuspend the cell pellet, forming a single-cell suspension. The suspension was then mixed with antibodies against CD45-APC (BioLegend) and CD34-APC (BioLegend) and incubated on ice in the dark for 30 min. The reaction was terminated by adding PBS with 10% FBS, and the samples were centrifuged at 4 °C and 1 000 r/min for 5 min. Next, 500 μL of PBS was added to resuspend the cells, and negative selection was performed using a flow cytometer (Becton, Dickinson and Company). The sorted BMSCs were seeded in suitable culture dishes and cultured in a complete medium. The medium was changed every three days until the cells reached 80% confluence, after which the cells were passaged. FACS analysis revealed that these cells were homogeneously positive for the mesenchymal markers CD90 (CD90-PE Ab, BioLegend), CD44 (CD44-PE Ab, BioLegend) and CD29 (CD29-APC Ab, BioLegend) and the stem cell marker Sca-1 (CD34-BV421 Ab, BioLegend) but negative for the hematopoietic markers CD34 (CD34-APC Ab, BioLegend) and CD45 (CD45-APC Ab, BioLegend). The morphology of the BMSCs, which adopted a fusiform, homogenous fibroblastic morphology, was observed under a light microscope. Further differentiation assays showed that these cells could readily differentiate into osteoblasts and adipocytes (Fig. S7). These cells expressed MSC markers and multilineage differentiation potential, aligning with the definition of BMSCs.^89^ The BMSCs used in this study were obtained from the third passage.

For the induction of senescence models and in vitro transfection experiments, mouse BMSCs were purchased from Cyagen Biosciences and maintained in DF12 medium supplemented with 10% FBS at 37 °C and 5% CO_2_. A 1,25(OH)_2_D solution was prepared by dissolving the powder in anhydrous ethanol (Aladdin). After the cells were seeded in a new plate and incubated for 24 h, the following substances were added to the respective groups under light-protected conditions: alcohol + 10% BSA (Veh group), 100 nmol/L 1,25(OH)_2_D + 10% BSA (1,25VD group), alcohol + 50 μmol/L PA (PA group), 100 nmol/L 1,25(OH)_2_D + 50 μmol/L PA (PA + 1,25VD group), and 1 mmol/L NAC (AbMole) + 50 μmol/L PA (NAC + 1,25VD group). The cells were incubated under light-protected conditions at 37 °C and 5% CO_2_ for 24 h. After digestion using TrypLE, the cells were transferred to a new plate for 12 h of subsequent experimental analysis.

### Preparation of a PA solution

A PA solution used to induce the senescence model was prepared according to previous methods.^90^ Briefly, 0.6 g of fatty acid-free BSA (Solarbio) was dissolved in 3 mL of double-distilled water to prepare a 20% fatty acid-free BSA solution. A 0.2% NaOH solution was prepared by dissolving 0.02 g of NaOH in 10 mL of ultrapure water. Then, 0.015 36 g of PA (Sigma) was added to 3 mL of the NaOH solution, and the mixture was saponified in a water bath at 75 °C for 30 min to obtain a 20 mmol/L palmitate saponification solution, which was mixed with the 20% fatty acid-free BSA solution at a 1:1 ratio to obtain a 10 mmol/L PA solution for subsequent experiments. A 10% BSA solution was prepared by mixing the 20% fatty acid-free BSA solution with an equal volume of the 0.2% NaOH solution.

### RNA isolation and real‐time RT‒qPCR

Total RNA was extracted from cultured MSCs using NucleoZOL reagent (MACHEREY‑NAGEL) according to the manufacturer’s instructions. Complementary DNA (cDNA) was synthesized using the PrimeScript RT Reagent Kit (TaKaRa). Real-time RT‒qPCR was carried out using a Roche Real-time System. Ppia was amplified at the same time to normalize gene expression. Each experiment was repeated three times to determine differences in relative gene expression. The sequences of the PCR primers used in this study are shown in Table S1.

### RNA-seq

Following the RNA extraction process described earlier, mRNA was fragmented, labeled, and reverse transcribed into cDNA, which was then purified with the TruSeq RNA Sample Prep Kit (Vazyme) according to the manufacturer’s instructions. The concentration of the resulting cDNA was measured using Qubit fluorometric quantification (Thermo Fisher). Subsequently, the samples were sent to Novogene Biotech Co., Ltd., for sequencing. The raw sequencing data were subjected to quality control analysis, intergroup comparisons, and the generation of gene expression heatmaps and volcano plots using R language.

Next, genes whose expression was significantly upregulated or downregulated in the HFD group compared to that in the ND group at log_2_FC > 1 and *P* < 0.05 were chosen for further analyses. Metascape was used to perform GO enrichment analysis of all the differentially expressed genes. Additionally, GSEA software was used to analyze whether specific gene sets, namely, the VDR PATHWAY (C2) and CELLULAR SENESCENCE (C5) gene sets, exhibited differential expression between the groups.^91,92^

### ATAC-seq

The viability of the digested cells was evaluated using trypan blue (Gibco), with the viability threshold set at 85% or higher. A total of 5 × 10^4^ cells per sample were isolated for ATAC library preparation using the TruePrep^TM^ DNA Library Prep Kit V2 for Illumina® (Vazyme) and the TruePrep^TM^ Index Kit V2 for Illumina® (Vazyme). The cells were washed once with 50 μL of cold PBS and centrifuged at 500 × *g* for 5 min at 4 °C. The cell pellets were resuspended in 50 µL of ice-cold lysis buffer and incubated on ice for 10 min. The cells were then centrifuged at 4 °C and 500 × *g* for 5 min to collect the nuclei. Next, 20 µL of a transposase reaction mixture was added, followed by transfer of the suspension to a new PCR tube containing the nuclear pellet. The reaction was carried out at 37 °C for 30 min. The resulting product was purified using DNA purification and magnetic bead purification, and the supernatant was transferred to a new PCR tube. PCR amplification was performed using double-ended adapter primers, PPM, DNA polymerase, and a TAB solution. The product was subjected to quality control through Qubit concentration measurements and fragment distribution analysis using the Bioptic Qsep 100 analysis system. The samples were subsequently sent for sequencing (conducted by Novogene Biotech Co., Ltd.).

After quality control, initial sequence alignment, and the removal of duplicate and mitochondrial sequences, MACS2 was used for peak calling, followed by DESeq2 for intergroup comparisons. Peaks were annotated using ChIPseeker to determine their genomic positions and nearby genes, and volcano plots were generated. The distribution of the ATAC-seq peaks was visualized using deepTools, and all the data were visualized using Integrated Genomic Viewer (IGV) software. Further analysis focused on genes showing differential chromatin accessibility with an absolute log_2_FC > 1 and *P* < 0.05. Metascape was used to perform GO enrichment analysis of all the differentially accessible genes. Motif analysis was conducted using HOMER.

### Induction of osteogenic and adipocyte differentiation

To induce osteogenic differentiation, BMSCs were seeded at a density of 1 × 10^4^ cells per well in a 48-well plate and cultured in DF12 medium supplemented with 10% FBS. When the cell density reached 70%-80%, the medium was replaced with an osteogenic differentiation induction medium. During the first week, the medium was replaced with a fresh mineralization induction medium every 3 days, and during the second week, the medium was replaced every 2 days. After 9 days of osteogenic differentiation induction, RNA was extracted for the detection of osteogenic-related gene expression. After 21 days of osteogenic differentiation induction, the original culture medium was removed, and the cells were washed three times with PBS. Then, 4% paraformaldehyde was added for fixation at room temperature for 15 min, followed by three washes with PBS. The cells were stained with a 1% alizarin red solution (Cyagen Biosciences) for 15 min and then washed three times with ddH_2_O. Finally, the cells were observed and photographed by light microscopy (Leica). To quantify the ARS staining results, we analyzed the mineralized area relative to the total area by using ImageJ software (National Institutes of Health). An osteogenic differentiation induction medium was purchased from Cyagen Biosciences.

To induce adipocyte differentiation, BMSCs were seeded at a density of 1 × 10^4^ cells per well in a 48-well plate and cultured in a DF12 medium containing 10% FBS. When the cell density reached 70–80%, the medium was replaced with adipocyte differentiation medium (Cyagen Biosciences) according to the manufacturer’s instructions. After 9 days of differentiation, the cells were fixed in 4% paraformaldehyde phosphate buffer for 20 min and washed with phosphate-buffered saline (PBS). An Oil Red O solution (Cyagen Biosciences) was added, and the samples were incubated for 15 min and washed with PBS.

### Senescence‐associated‐β‐galactosidase (SA-β-gal) staining

BMSCs were seeded at a density of 1 × 10^5^ cells per well in a 6-well plate and incubated for 24 h, after which the original culture medium was removed, and the cells were washed three times with PBS. Following the instructions of an SA-β-gal assay kit (Beyotime Biotechnology), the cells were fixed at room temperature for 15 min, followed by three washes with PBS. A working solution was prepared and used for staining, and the cells were observed and photographed by light microscopy.

### CCK8 assay

BMSCs were seeded at a density of 1 × 10^3^ cells per well in a 96-well plate and cultured in DF12 medium containing 10% FBS. On the 1st, 3rd, 5th, 7th, 9th, and 11th days after seeding, the culture medium was replaced with a mixture of 100 μL of culture medium and 10 μL of CCK8 solution (YEASEN) under light-protected conditions. The cells were incubated at 37 °C and 5% CO_2_. The absorbance of each well at 450 nm was measured with a microplate reader (BioTek). For each group, triplicate wells were used, and the experiment was repeated at least three times.

### EdU staining

After BMSCs were seeded at a density of 1 × 10^5^ cells per well in a 6-well plate and incubated for 24 h, EdU (Beyotime Biotechnology) was added to the culture medium to a final concentration of 10 μM. The cells were then incubated at 37 °C and 5% CO_2_ for an additional 48 h. Afterward, the original culture medium was removed, and the cells were fixed, permeabilized, treated with reaction solution, and finally stained. The samples were observed and imaged by fluorescence microscopy (Leica).

### Immunocytochemical staining

For immunocytochemical staining, cultured cells were fixed in 4% (vol/vol) paraformaldehyde for 30 min at room temperature, permeabilized in PBST (1x PBS, 0.2% Triton X-100) for 10 min at room temperature, and incubated in sheep serum for blocking. Primary antibodies were incubated overnight at 4 °C, followed by incubation with secondary antibodies overnight at 4 °C. The primary antibodies used in this study included anti-p21 (BD Pharmingen) at a dilution of 1:100, anti-γH2A.X (Abcam) at a dilution of 1:500, and anti-VDR (Santa Cruz Biotechnology) at a dilution of 1:50. Alexa Fluor-coupled secondary antibodies were used (Cell Signaling Technology) at a dilution of 1:500.

### Western blot

Proteins were extracted from the BMSCs of each group of mice or from in vitro samples. The cytoplasmic and mitochondrial proteins were extracted using the Cytoplasmic and Mitochondrial Protein Extraction kit (Beyotime Biotechnology), according to the manufacturer’s instructions. Immunoblotting was conducted as previously described.^93^ Primary antibodies against P21 (BD Pharmingen) at 1:500, VDR (Santa Cruz Biotechnology) at 1:500, SOD1 (Santa Cruz Biotechnology) at 1:1 000, SOD2 (Santa Cruz Biotechnology) at 1:1 000, cytochrome C (Zen-Bioscience) at 1:500, AIF (Zen-Bioscience) at 1:500, cleaved caspase-3 (Zen-Bioscience) at 1:500 and Vinculin (Zen-Bioscience) at 1:2 000 were used. Immunoreactive bands were visualized with enhanced chemiluminescence (ECL) (Epizyme Biotech) and analyzed with a ChemiDoc^TM^ MP imaging system (Bio-Rad).

### Measurement of intracellular ROS levels

Intracellular ROS levels were determined using the oxidation-sensitive fluorescent probe DHE (KeyGEN) according to the manufacturer’s instructions. BMSCs were seeded at a density of 5 × 10^4^ cells per well in a 24-well plate and cultured in DMEM supplemented with 10% FBS for 24 h. Afterward, DHE was added to the medium to a final concentration of 10 µmol/L, and the cells were incubated at 37 °C and 5% CO_2_ for 30 min. The adherent cells were detached into single cells using TrypLE, followed by centrifugation at 1 000 r/min for 5 min and removal of the supernatant. The cells were resuspended in 200 μL of PBS and analyzed by flow cytometry.

### Measurement of SOD activity

BMSCs were seeded at a density of 3 × 10^5^ cells per well in a 12-well plate and cultured in a culture medium for 24 h. Afterward, the cells were scraped using a cell scraper and transferred to a centrifuge tube. Cold PBS was added, and the cells were sonicated on ice to ensure complete cell lysis. Afterward, the mixture was centrifuged at 4 °C and 12 000 × *g* for 5 min, after which the SOD activity was measured with a Total Superoxide Dismutase Assay Kit (Beyotime Biotechnology). The samples were prepared according to the manufacturer’s instructions and analyzed with a microplate reader.

### Luciferase activity assay

Using mouse genomic DNA as a template, the *Sod2* promoter region (−200 to +100) was amplified via PCR, which yielded two PCR products. One product contained the predicted VDR-binding site, “CAGGGTCA”, while the other product contained the mutation site, “ACTCCCGC”. These two PCR products were subsequently inserted into the GV238 vector, which contains the firefly luciferase gene, to generate the Sod2-GV238 and Sod2mut-GV238 mutant plasmids, respectively (Fig. S4A). All the abovementioned plasmids were synthesized by GENECHEM.

BMSCs were seeded at a density of 1 × 10^4^ cells per well in a black, opaque 96-well fluorescence plate. After 24 h, Lipofectamine 2000 (Thermo Fisher Scientific) was used to cotransfect the cells with a Renilla luciferase plasmid (internal control), and either the GV238 empty vector (the GV238 vector without the *Sod2* promoter region), the Sod2-GV238 plasmid, or the Sod2mut-GV238 mutant plasmid for 12 h. The medium was then replaced with culture medium supplemented with either 10^−7^ mol/L 1,25(OH)_2_D or an equal volume of ethanol for another 24 h. The luciferase activity was ultimately analyzed using a dual-luciferase assay kit (Promega Corporation, USA) following the manufacturer’s instructions.

### ELISA

BMSCs derived from HFD-fed and ND-fed mice were seeded in six-well plates at a density of 1 × 10^5^ cells per well and incubated for 72 h. The levels of IL6 in the cell supernatants were quantified with commercially available ELISA kits (Cusabio, Wuhan, China) following the manufacturer’s directions.

### RNA interference

To suppress endogenous levels of SOD2, BMSCs were transfected with siRNAs targeting SOD2 (RiboBio) using Lipofectamine 2000 (Thermo Fisher Scientific) following the manufacturer’s protocol. For the analysis of the resulting protein expression levels, the cells were lysed for Western blot analysis at specified time points after transfection.

The cells were initially seeded in antibiotic-free growth medium 24 h before transfection. The transfection solution was prepared as follows: Lipofectamine 2000 at a concentration of 1 mg/mL was diluted to 30 µg/mL in Opti-MEM (31985, Gibco) and mixed for 15 min. Simultaneously, 20 × 10^−6^ mol/L solutions of each siRNA were diluted to 1 × 10^−6^ mol/L in a separate aliquot of Opti-MEM. The two solutions were then combined at a 1:1 ratio by volume and incubated for an additional 15 min at room temperature. Subsequently, the final mixture was introduced into the cell culture medium without antibiotics to a final siRNA concentration of 100 × 10^−9^ mol/L and Lipofectamine 2000 concentration of 3 µg/mL. The cells were incubated with siRNA for 24 h at 37 °C with 5% CO_2_. Cells treated solely with Lipofectamine 2000 served as the negative control group.

### Catalase activity assay

Proteins were extracted from the BMSCs of ND- or HFD-fed mice. The protein concentration was measured using a BCA protein assay kit (CWBIO). The supernatant was subjected to catalase activity analysis with a Catalase Assay Kit (Beyotime) according to the manufacturer’s instructions.

### Statistical analysis

All the data were presented as the mean ± SEM of at least three independent experiments. After normality testing, all the data were analyzed by two-tailed unpaired Student’s *t*-test, the Kruskal–Wallis test (nonparametric sample) or one-way ANOVA (parametric sample) followed by Dunn’s test (nonparametric sample) or Tukey’s post hoc test (parametric sample) as a post hoc test. All the statistical analyses were performed with GraphPad Prism software.

### Supplementary information

Multiomics profiling reveals VDR as a central regulator of mesenchymal stem cell senescence with a known association with osteoporosis after high-fat diet exposure

## Data Availability

The raw sequence data reported in this paper have been deposited in the Genome Sequence Archive (GSA: CRA012133 and CRA012134) of the National Genomics Data Center and are publicly accessible at https://ngdc.cncb.ac.cn/gsa.
