# Supplementary material for: Multiomics profiling reveals VDR as a central regulator of mesenchymal stem cell senescence with a known association with osteoporosis after high-fat diet exposure
Source: Int J Oral Sci. 2024 May 22;16:41. doi: 10.1038/s41368-024-00309-9 (PMC11111693; doi:10.1038/s41368-024-00309-9)
Supplement: Supplementary file 1 — Multiomics profiling reveals VDR as a central regulator of mesenchymal stem cell senescence with a known association with osteoporosis after high-fat diet exposure [file 41368_2024_309_MOESM1_ESM.pdf]

Multiomics profiling reveals VDR as a central regulator of mesenchymal stem cell senescence with a known association with osteoporosis after high-fat diet exposure

Jiayao Chen[1,2,3*], Shuhong Kuang[1,2,3*], Jietao Cen[1,2,3], Yong Zhang[1,2,3], Zongshan Shen[1,2,3], Wei Qin[1,2,3], Qiting Huang[1,2,3], Zifeng Wang[4], Xianling Gao[1,2,3], Fang Huang[1,2,3#], Zhengmei Lin[1,2,3#]

1. Hospital of Stomatology, Sun Yat-sen University, Guangzhou, China.
2. Guangdong Provincial Key Laboratory of Stomatology, Guangzhou, China.
3. Guanghua School of Stomatology, Sun Yat-sen University, Guangzhou, China.
4. Sun Yat-sen University Cancer Center; State Key Laboratory of Oncology in South China; Collaborative Innovation Center for Cancer Medicine; Guangzhou, China

[*]These authors contributed equally to this work.

[#] Corresponding authors:

Zhengmei Lin, Hospital of Stomatology, Guangdong Provincial Key Laboratory of Stomatology, Guanghua School of Stomatology, Sun Yat-Sen University, No. 56, Lingyuan West Road, Yuexiu District, Guangzhou, Guangdong 510055, China. E-mail: linzhm@mail.sysu.edu.cn

Fang Huang, Hospital of Stomatology, Guangdong Provincial Key Laboratory of Stomatology, Guanghua School of Stomatology, Sun Yat-Sen University, No. 56, Lingyuan West Road, Yuexiu District, Guangzhou, Guangdong 510055, China. E-mail: hfang@mail.sysu.edu.cn

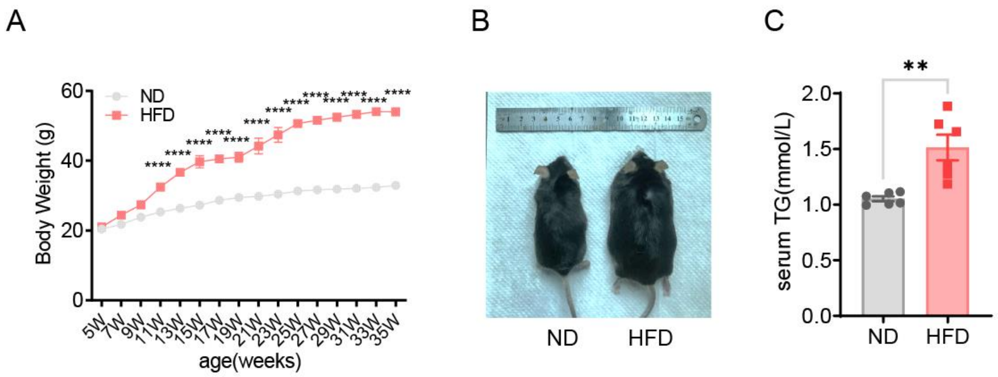

Figure S1. Body weight and the serum triglyceride concentration are altered in HFD-fed mice

(A) C57BL/6J mice were fed a high-fat diet (HFD) or normal diet (ND) beginning at 5 weeks of age, and changes in the body weight of the mice were detected regularly until 35 weeks of age. (B) Photographs of mice from each group after 30 weeks of dietary intervention. (C) Serum triglyceride concentrations of 35-week-old HFD-fed mice and ND-fed mice. N = 6, *$P < 0.05$, **$P < 0.01$, ***$P < 0.001$, ****$P < 0.0001$.

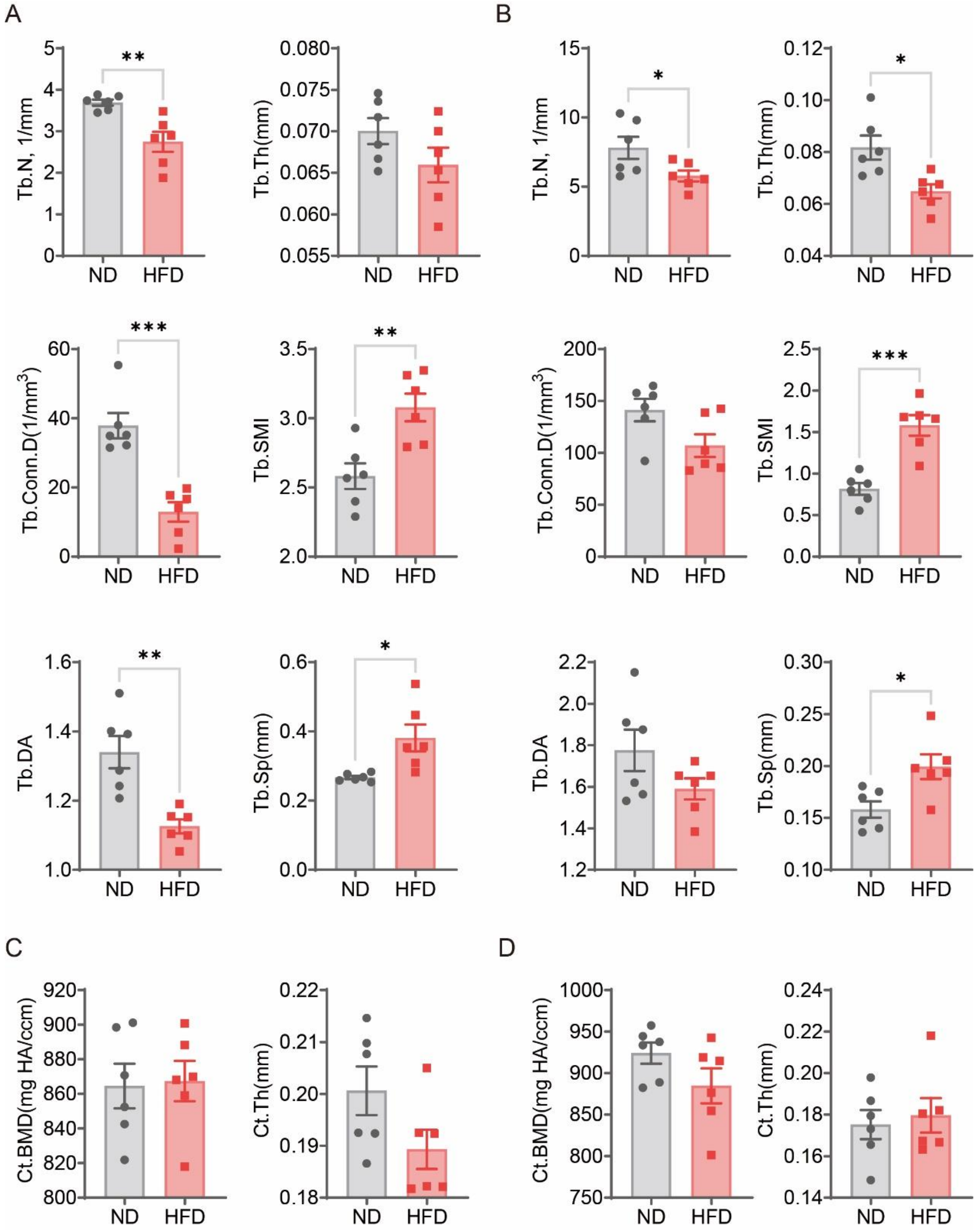

Figure S2. HFD feeding decreases the trabecular bone mass in the mandible and femur

(A) μCT analyses of the trabecular number (Tb.N), trabecular thickness (Tb.Th), trabecular connectivity density (Tb.Conn.D), structural model index (Tb.SMI), degree of anisotropy (Tb.DA) and trabecular spacing (Tb.Sp) in the distal femur metaphysis of HFD-fed mice and ND-fed mice. (B) Trabecular bone parameters of the first mandibular molar root furcation regions of HFD-fed mice and ND-fed mice were

determined by μCT. (C) μCT analyses of cortical BMD and cortical bone thickness (Ct.Th) at the femoral mid-diaphysis of HFD-fed mice and ND-fed mice were carried out. (D) μCT analyses of the cortical bone parameters in the first mandibular molar root furcation regions of HFD-fed mice and ND-fed mice were carried out. The data are shown as the mean ± SEM. N=6, *$P < 0.05$, **$P < 0.01$, ***$P < 0.001$.

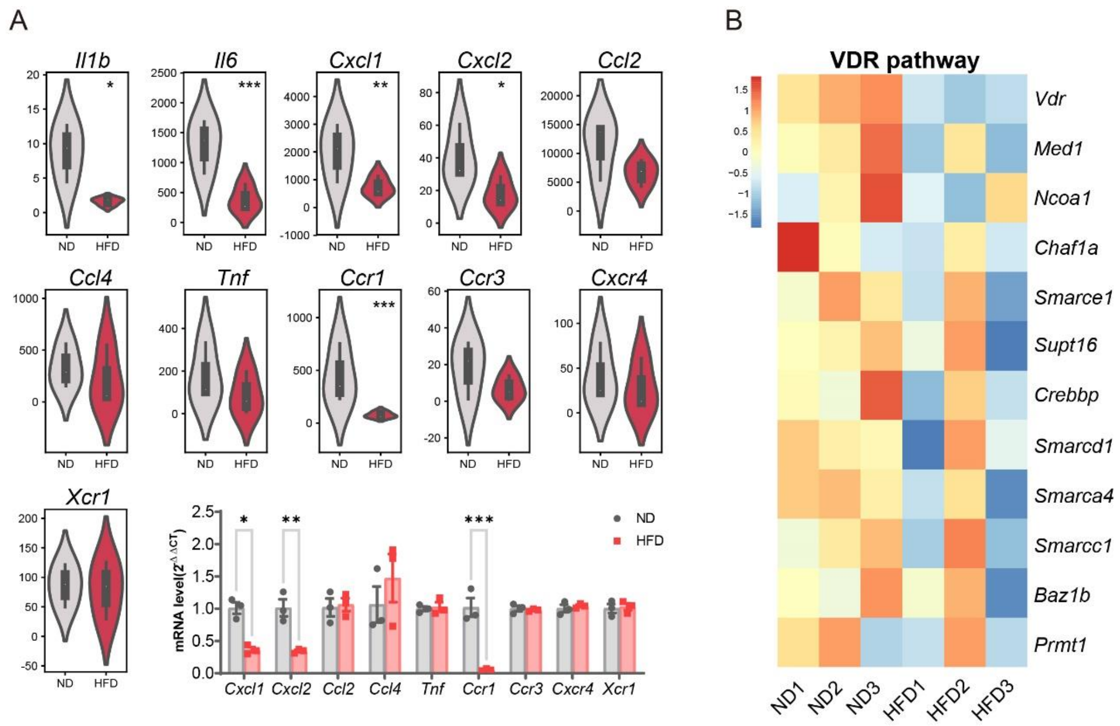

Figure S3. HFD feeding decreases the expression of certain proinflammatory genes in bone marrow stromal cells (BMSCs)

(A) Violin plots showing the expression levels of classic proinflammatory genes and chemokine receptor genes in BMSCs from the HFD-fed mice and the ND-fed mice. Violin plots show the medians, quartiles, and 95% confidence intervals. The expression of classic proinflammatory genes and chemokine receptor genes was determined by RT-qPCR, shown as the mean ± SEM. (B) Heatmap showing the downregulated expression of VDR pathway genes in the BMSCs from the HFD-fed mice. N = 3, $*P < 0.05$, $**P < 0.01$, $***P < 0.001$, $****P < 0.0001$.

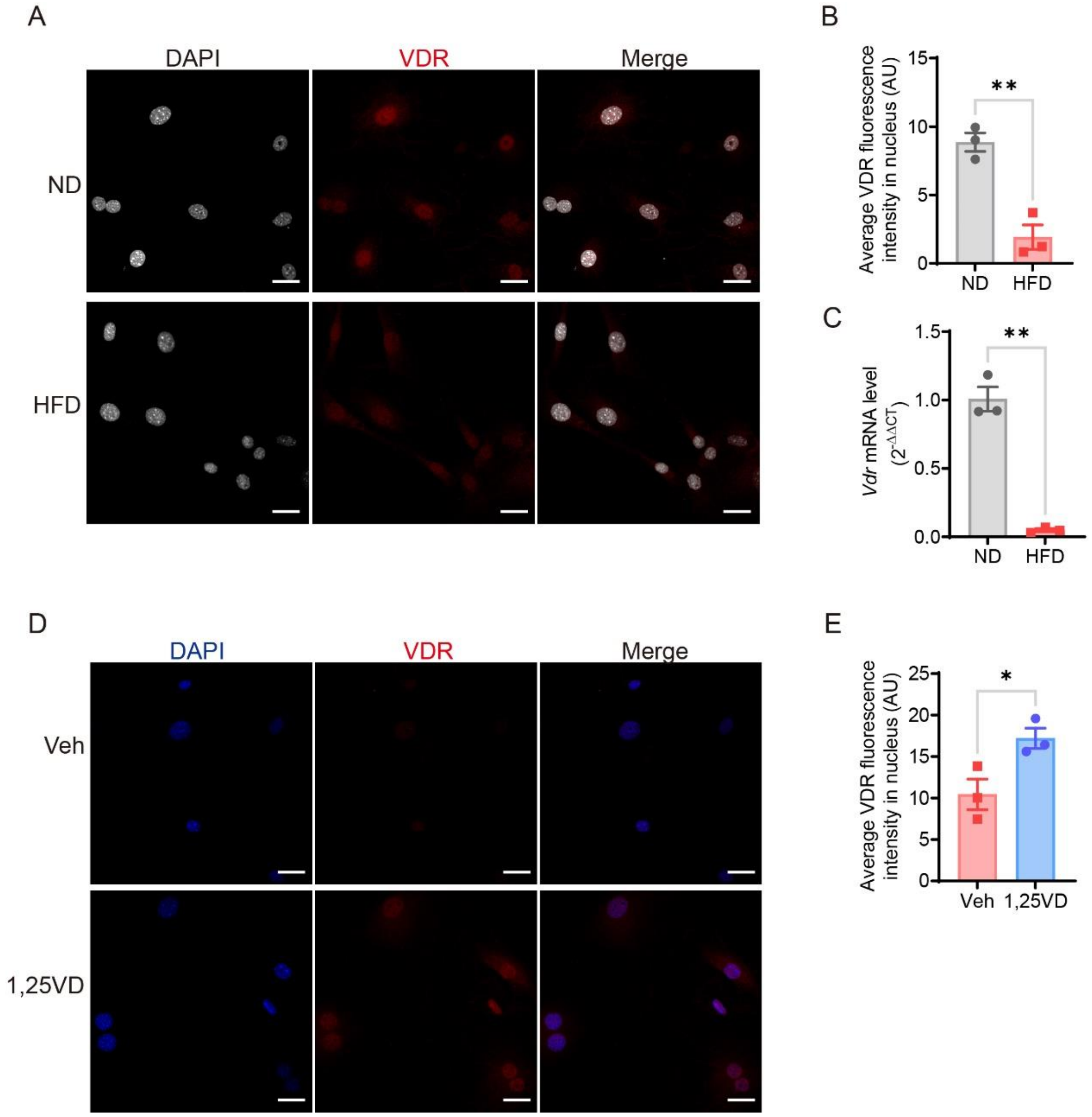

Figure S4. 1,25(OH)$_2$D reverses the decrease in the expression of VDR induced by a HFD in BMSCs

(A) The expression of the VDR protein in the BMSCs of HFD-fed mice or ND-fed mice was evaluated via immunofluorescence staining (scale bars = 20 μm). (B) VDR expression in the HFD group and ND group was quantified by immunofluorescence staining. (C) The transcription level of VDR in the BMSCs in each group was detected via RT–qPCR. (D) Immunofluorescence staining was carried out to assess the expression of VDR in BMSCs from the Veh group or 1,25VD group. (E) VDR expression in the Veh group and 1,25VD group was quantified by immunofluorescence staining. The data are shown as the mean ± SEM. N = 3, $*P < 0.05$, $**P < 0.01$.

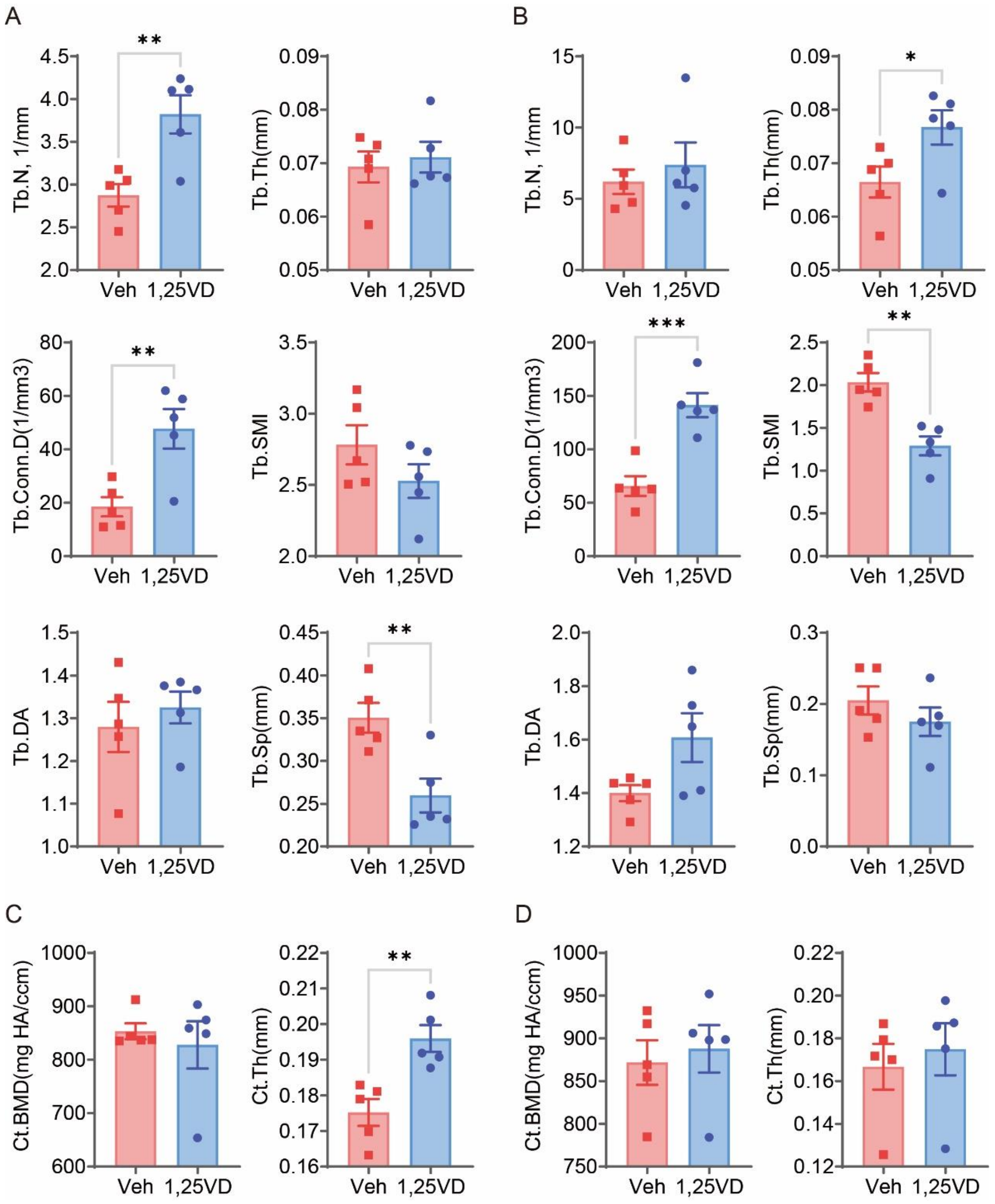

Figure S5. Upregulation of VDR signaling rescues bone loss induced by a HFD in the mandible and femur

(A) μCT analyses of the trabecular number (Tb.N), trabecular thickness (Tb.Th), trabecular connectivity density (Tb.Conn.D), structural model index (Tb.SMI), degree of anisotropy (Tb.DA) and trabecular spacing (Tb.Sp) in the distal femur metaphysis of the Veh group or 1,25VD group were carried out. (B) Trabecular bone parameters of the first mandibular molar root furcation regions of the Veh group or 1,25VD group were determined by μCT. (C) μCT analyses of cortical BMD and cortical bone

thickness (Ct.Th) at the femoral diaphysis in the Veh group or 1,25VD group were carried out. (D) μCT analyses of cortical bone parameters in the first mandibular molar root furcation regions of the Veh group or 1,25VD group were carried out. The data are shown as the mean ± SEM. N=5, **P* < 0.05, ***P* < 0.01, ****P* < 0.001.

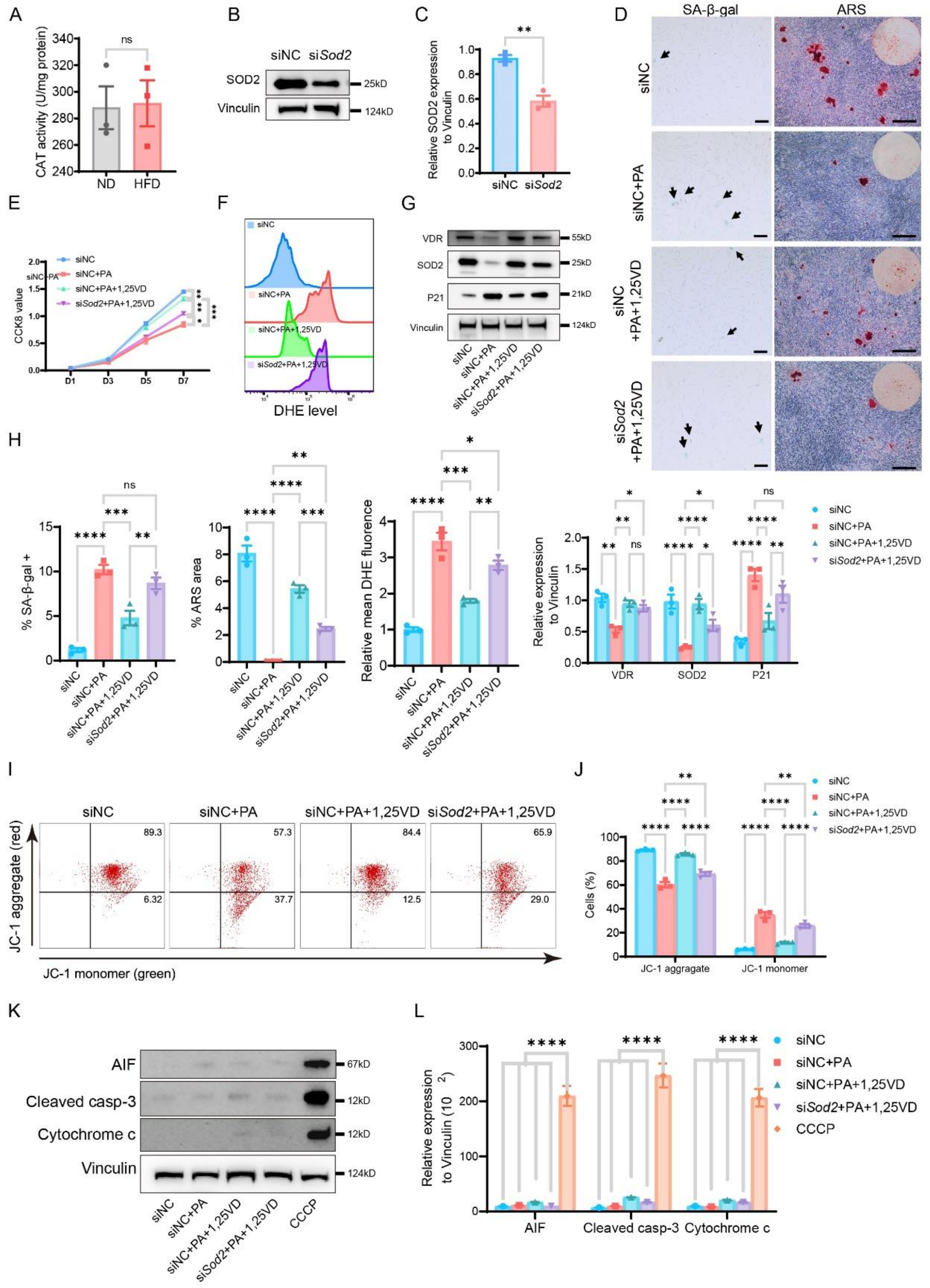

Figure S6. SOD2 deficiency impairs the anti-senescence effects of VDR activation in BMSCs

(A) CAT activity data show no significant difference in the CAT enzymatic activity of BMSCs between the HFD group and the ND group. (B-C) The expression of SOD2 in

the BMSCs treated with siRNA against *Sod2* (si*Sod2*) or nontargeting control siRNA (siNC) was determined via Western blot. (D-H) BMSCs were treated with si*Sod2* or siNC, followed by treatment with PA, PA+1,25VD or vehicle. A reduction in *Sod2* expression by siRNA impaired the ability of 1,25$(OH)_2$D to alleviate senescence (D, G), promote proliferation (E), decrease intracellular ROS levels (F) and increase the osteogenic differentiation capacity (D). The percentages of SA-β-gal-positive cells; ROS levels; VDR, p21 and SOD2 expression; and ARS staining of BMSCs from each group are shown in (H). The positive SA-β-gal cells were stained green (black arrow). (I-J) BMSCs were subjected to JC-1 staining. The proportion of JC-1 monomers (green) or JC-1 aggregates (red) was measured via flow cytometry. (K-L) There was no significant change in the cytosolic levels of apoptosis-inducing factor (AIF), cytochrome c or cleaved caspase-3 (cleaved casp-3) upon si*Sod2* transfection, as shown by Western blot analysis of cytosolic samples. Carbonyl cyanide m-chlorophenyl hydrazone (CCCP) was used as positive control. The data are shown as the mean ± SEM. N = 3, *$P < 0.05$, **$P < 0.01$, ***$P < 0.001$, ****$P < 0.0001$.

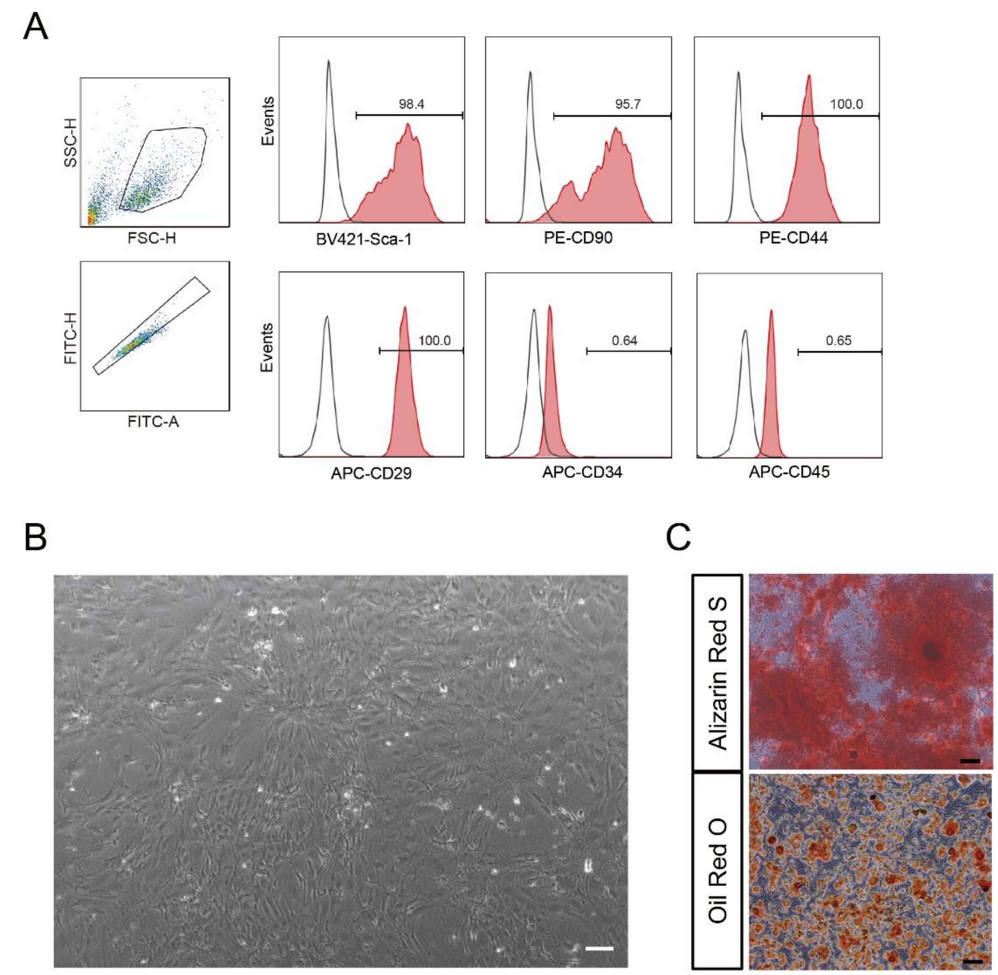

Figure S7. Authentication of BMSCs.

(A) Flow cytometry was used to examine the cell-surface antigen profile of BMSCs, and the results showed that the cells isolated from bone marrow expressed mesenchymal markers (CD90, CD44, and CD29) and the stem cell marker Sca-1 without hematopoietic cell line markers (CD34 and CD45). (B) Microscopy image of BMSCs at 100x magnification (scale bar = 50 μm). (C) Alizarin red staining and oil red O staining were used to detect the osteogenic and adipogenic differentiation capacities of BMSCs (scale bars = 50 μm).

Table S1. Sequences of the primers used for RT–qPCR

Name	Sequence (5’-3’)
CDKN1A(p21)	CCTGGTGATGTCCGACCTG
	CCATGAGCGCATCGCAATC
CDKN2A(p16)	AACTCTTTCGGTCGTACCCC
	GCGTGCTTGAGCTGAAGCTA
TRP53(p53)	CCCCTGTCATCTTTTGTCCCT
	AGCTGGCAGAATAGCTTATTGAG
COL1A1	CTGGCGGTTCAGGTCCAAT
	TTCCAGGCAATCCACGAGC
OCN	CTGACCTCACAGATGCCAAGC
	TGGTCTGATAGCTCGTCACAAG
ALP	CCAACTCTTTTGTGCCAGAGA
	GGCTACATTGGTGTTGAGCTTTT
BSP	ATGGAGACGGCGATAGTTCC
	CTAGCTGTTACACCCGAGAGT
OSX	TCCCTGGATATGACTCATCCCT
	CCAAGGAGTAGGTGTGTTGCC
VDR	GAATGTGCCTCGGATCTGTGG
	ATGCGGCAATCTCCATTGAAG
HMOX1	GATAGAGCGCAACAAGCAGAA
	CAGTGAGGCCCATACCAGAAG
SOD1	AACCAGTTGTGTTGTCAGGAC
	CCACCATGTTTCTTAGAGTGAGG
SOD2	CAGACCTGCCTTACGACTATGG
	CTCGGTGGCGTTGAGATTGTT
CAT	AGCGACCAGATGAAGCAGTG
	TCCGCTCTCTGTCAAAGTGTG
NRF2	TAGATGACCATGAGTCGCTTGC
	GCCAAACTTGCTCCATGTCC
FOXO3	GCAAGCCGTGTACTGTGGA
	CGGGAGCGCGATGTTATCC
PRDX4	CTCAAACTGACTGACTATCGTGG
	CGATCCCCAAAAGCGATGATTTC

CXCL1	CTGGGATTCACCTCAAGAACATC
	CAGGGTCAAGGCAAGCCTC
CXCL2	CCAACCACCAGGCTACAGG
	GCGTCACACTCAAGCTCTG
CCL2	TTAAAAACCTGGATCGGAACCAA
	GCATTAGCTTCAGATTTACGGGT
CCL4	TTCCTGCTGTTTCTCTTACACCT
	CTGTCTGCCTCTTTTGGTCAG
TNF	CCCTCACACTCAGATCATCTTCT
	GCTACGACGTGGGCTACAG
CCR1	CTCATGCAGCATAGGAGGCTT
	ACATGGCATCACCAAAAATCCA
CCR3	TCAACTTGGCAATTTCTGACCT
	CAGCATGGACGATAGCCAGG
CXCR4	GAAGTGGGGTCTGGAGACTAT
	TTGCCGACTATGCCAGTCAAG
XCR1	CTCAGCCTTGTGGGTAACAGC
	ACAGGCAGTAGACAGGAGAAC
PPIA	GAGCTGTTTGCAGACAAAGTTC
	CCCTGGCACATGAATCCTGG
